# Quantitative trait loci in hop (*Humulus lupulus* L.) reveal complex genetic architecture underlying variation in sex, yield and cone chemistry

**DOI:** 10.1186/1471-2164-14-360

**Published:** 2013-05-30

**Authors:** Erin L McAdam, Jules S Freeman, Simon P Whittock, Emily J Buck, Jernej Jakse, Andreja Cerenak, Branka Javornik, Andrzej Kilian, Cai-Hong Wang, Dave Andersen, René E Vaillancourt, Jason Carling, Ron Beatson, Lawrence Graham, Donna Graham, Peter Darby, Anthony Koutoulis

**Affiliations:** 1 School of Plant Science, University of Tasmania, Private Bag 55, Hobart TAS 7001, Australia; 2 Faculty of Science, Health, Education and Engineering, University of the Sunshine Coast, Locked Bag 4, Maroochydore 4558 QLD, Australia; 3 Hop Products Australia, 26 Cambridge Road, Bellerive 7018 TAS, Australia; 4 The New Zealand Institute for Plant & Food Research Limited, Private Bag 11 600, Palmerston North 4442, New Zealand; 5 Agronomy Department, Centre for Plant Biotechnology and Breeding, Biotechnical Faculty, University of Ljubljana, Jamnikarjeva 101, Ljubljana 1000, Slovenia; 6 Slovenian Institute of Hop Research and Brewing, Cesta Zalskega Tabora 2, Zalec 3310, Slovenia; 7 Diversity Arrays Technology Pty Ltd, PO Box 7141, Yarralumla 2600ACT, Australia; 8 Department of Horticulture, Qingdao Agricultural University, Qingdao 266109, China; 9 The New Zealand Institute for Plant & Food Research Limited, Old Mill Road, Motueka 7120, New Zealand; 10 Wye Hops Ltd., China Farm, Upper Harbledown, Canterbury, Kent CT2 9AR, UK

**Keywords:** Linkage map, Transferable marker, Diversity arrays technology DArT, Pleiotropy, Sex-linked marker, Dry cone weight, Plant secondary metabolite, Hop acid, Essential oil

## Abstract

**Background:**

Hop (*Humulus lupulus* L.) is cultivated for its cones, the secondary metabolites of which contribute bitterness, flavour and aroma to beer. Molecular breeding methods, such as marker assisted selection (MAS), have great potential for improving the efficiency of hop breeding. The success of MAS is reliant on the identification of reliable marker-trait associations. This study used quantitative trait loci (QTL) analysis to identify marker-trait associations for hop, focusing on traits related to expediting plant sex identification, increasing yield capacity and improving bittering, flavour and aroma chemistry.

**Results:**

QTL analysis was performed on two new linkage maps incorporating transferable Diversity Arrays Technology (DArT) markers. Sixty-three QTL were identified, influencing 36 of the 50 traits examined. A putative sex-linked marker was validated in a different pedigree, confirming the potential of this marker as a screening tool in hop breeding programs. An ontogenetically stable QTL was identified for the yield trait dry cone weight; and a QTL was identified for essential oil content, which verified the genetic basis for variation in secondary metabolite accumulation in hop cones. A total of 60 QTL were identified for 33 secondary metabolite traits. Of these, 51 were pleiotropic/linked, affecting a substantial number of secondary metabolites; nine were specific to individual secondary metabolites.

**Conclusions:**

Pleiotropy and linkage, found for the first time to influence multiple hop secondary metabolites, have important implications for molecular selection methods. The selection of particular secondary metabolite profiles using pleiotropic/linked QTL will be challenging because of the difficulty of selecting for specific traits without adversely changing others. QTL specific to individual secondary metabolites, however, offer unequalled value to selection programs. In addition to their potential for selection, the QTL identified in this study advance our understanding of the genetic control of traits of current economic and breeding significance in hop and demonstrate the complex genetic architecture underlying variation in these traits. The linkage information obtained in this study, based on transferable markers, can be used to facilitate the validation of QTL, crucial to the success of MAS.

## Background

Hop is an important agronomic commodity, used mainly in the brewing industry. Rich in secondary metabolites, hop cones (female inflorescences) are an essential raw ingredient in beer, contributing the distinctive bitterness, flavour and aroma, as well as preservative activity [1-4]. Traditional breeding methods have made significant progress in increasing the yield of hops and altering hop secondary metabolite profiles to improve bittering, flavour and aroma potential. Traditional breeding in hop is based on phenotypic selection of superior genotypes within segregating progenies obtained from crosses. As is the case with many perennial crops, this is a complex and lengthy process. Molecular breeding methods, such as marker assisted selection (MAS), have the potential to complement conventional phenotypic-pedigree based selection methods by providing a sophisticated, direct and precise selection system, with the capacity for higher throughput [5-8]. The successful application of MAS relies on understanding the genetic architecture underlying variation in the phenotype of traits [9]. More specifically, MAS requires the identification of molecular markers closely associated with trait variation [10]. Among other techniques, quantitative trait loci (QTL) analysis can be used to identify marker-trait associations. The genetic information acquired in QTL analysis, such as the number, location and magnitude of effects of genetic regions associated with a trait, also contributes significantly to the overall understanding of trait heritability [9].

Linkage maps are a prerequisite for QTL analysis and must be of high quality to ensure accuracy, resolution and reproducibility in the QTL identified [11]. When constructing linkage maps, transferrable markers, such as microsatellite or Diversity Arrays Technology (DArT) markers, are preferable to less transferable markers, such as amplified fragment length polymorphism (AFLP) and random amplified polymorphic DNA (RAPD) markers, as they are easily employed in different mapping populations, thus facilitating direct comparison between maps and QTL verification [12]. This validation of QTL is crucial for the broad success of MAS, since QTL can be restricted to the pedigree, environment and ontogenetic stage in which they were discovered [13]. In hop, only a small number of QTL studies have been undertaken. Linkage maps have been constructed in four pseudo-testcross populations [14-17] and with the exception of one [17], these are dominated by AFLP and RAPD markers, with a small proportion of transferable markers [14-16]. A few studies have identified QTL [14,16-18] and other marker-trait associations [19-26]; however, only twelve traits have been examined and no validation has so far been reported. Further linkage analyses are required to gain a better understanding of loci influencing important hop traits and ultimately to enable MAS for these traits across different breeding programs. Three key areas of economic significance targeted for the genetic improvement of hop are: expedited plant sex identification, increased yield capacity and improved secondary metabolite profiles. The identification of QTL related to these targets would aid hop breeding programs in their efforts to meet the needs of the brewing industry.

Hop is a predominantly dioecious species, with male and female flowers produced on separate plants. Only female plants have economic value, producing cones containing lupulin, the secondary metabolite-packed resin, which is the commercial product. Hop has a genome size of 2.8 pg (2.7 Gbp) [27], similar to the average for all eudicots (2.8 pg) [28]. Cytogenetic studies have demonstrated that hop is diploid (2*n* = 2*x* = 20), with nine autosomal (A) chromosome pairs and two sex chromosomes (X and Y) [21,29-31]. Sex determination is dependent on an X/A balance, a system found in a few other plant genera, such as *Cannabis* and *Rumex*[32]. A ratio of the number of X chromosomes to the number of sets of autosomes of 1.0 gives rise to a female plant; a ratio of 0.5 gives rise to a male plant; and intermediate ratios give rise to monoecious plants (male and female flowers produced on the same plant) [31,32]. Under the X/A balance system for determination of sex phenotype, the Y chromosome is not essential to the development of the male phenotype, but is required for pollen maturation [32]; sex expression may be regulated by genes on the autosomes or may be X-linked. Definitive determination of the sex phenotype requires two seasons of growth. Sex determination at the seedling stage, using molecular markers, would drastically reduce hop breeding program costs and optimise utilisation of available land. A number of sex-linked molecular markers have been identified in hop, including RAPD markers [19,23], inter simple sequence repeat (ISSR) markers [20], microsatellites [22] and cytogenetic markers [21]. Most markers are associated with the Y chromosome and are thus linked to maleness. However, the use of these markers has had mixed success in breeding, as the majority remain unverified across second or multiple populations. For some of these markers there is also evidence for incomplete linkage to the male sex [33]. The best described male sex-linked marker is a microsatellite, HLAGA7, being completely linked to the male sex in two Slovenian populations and on a representative sample of male hop genotypes [22]. Although HLAGA7 provides a robust sex-linked marker for use as a screening tool in hop breeding programs, further research may detect additional polymorphic loci located on autosomes which affect gender in a broader range of genetic material.

Increasing the yield of commercial product is one of the main goals of hop breeding programs and is largely based on two methods: (i) directly increasing the content of commercially important secondary metabolites (such as hop acids, essential oils and flavonoids [34,35]) in hop cones; or (ii) indirectly increasing secondary metabolite yield, by increasing flower number and subsequently cone production. In most cultivated plant species, the inheritance of yield is complex; influenced by a multitude of integrated physiological and biochemical processes, each with their own genetic basis [36,37] and hop is no exception [38-40]. Yield may also be influenced by a number of environmental factors, including water supply [41-44], nutrient availability, day length [41,45], irradiance [43,44], temperature [43,44], agricultural practice [46] and infestation of pests and diseases [47-50]. The identification of QTL influencing yield and their utilisation for MAS would greatly assist breeding for increased hop yield, by eliminating confounding environmental influences as well as allowing assessments of yield potential at the seedling stage, several years before maximal cone yields, or in non-yielding male plants. An earlier study has identified putative QTL for cone yield traits, including microsatellite and AFLP markers linked to cone harvest index and dry cone weight [16]. However, given the genetic complexity of yield in other plants, there are potentially further regions of the genome associated with yield traits for which QTL could be identified.

The secondary metabolite profile of hop is diverse, consisting of three broad chemical groups: (i) hop acids (or prenylated polyketides), divisible into the subgroups α-acids and β-acids; (ii) essential oils (both terpenoid and oxygenated compounds); and (iii) polyphenols [51]. Alpha-acids impart the characteristic bitter taste to beer, while essential oils are responsible for flavour and aroma [3]. Beta-acids also contribute to beer bitterness, as well as functioning as preservative agents, possessing anti-microbial properties [1-4]. The influence of polyphenols in beer brewing are not thoroughly understood, but several polyphenol compounds have been found to have potential pharmaceutical applications, particularly 8-prenylnarigenin as a phytoestrogen [52] and xanthohumol as a cancer chemopreventative agent [53]. Secondary metabolites accumulate in high concentrations in lupulin glands, which are peltate glandular trichomes found in great density on the bracteoles in hop inflorescences (cones) [54,55]. There is evidence to show that the lupulin glands may also be involved in the biosynthesis of the secondary metabolites [56]. In hop, differences in secondary metabolite composition are genotype-specific, with different cultivars having characteristic secondary metabolite profiles and subsequently unique bittering potentials and distinct flavour profiles [57,58]. Chemical profiles also vary with the maturation of the hop cone [59,60] and the effects of environmental stimuli. The secondary metabolite profile of kiln-dried hop cones consists of up to 30% hop acids, dominated by humulones (α-acid) and lupulones (β-acid) [51,61]. Polyphenols and tannins comprise 3 to 6% of the hop cone weight, while essential oils are found at levels between 0.5 and 5.0 ml per 100 g [51,61,62]. Typically, 90% of essential oils are terpenoids, dominated by myrcene, humulene, caryophyllene and farnesene [51,61,62]. The composition of hop essential oil is diverse, with around 500 compounds currently identified and suggestions that around 1000 compounds might be present [63]. The biosynthesis of secondary metabolites is complex and not completely understood, with many of the enzymes involved yet to be identified. The three secondary metabolite chemical classes present in hop are derived from pathways of terpene metabolism, following the 2-C-methylerythritol 4-phosphate (MEP) pathway [64,65]. The biosynthesis of these hop secondary metabolites involve common precursors, including isopentenyl pyrophosphate (IPP), dimethylallyl diphosphate (DMAPP) and malonyl coenzyme A [64,66-68]. Consequently, the synthesis of the different components may be competitive and common loci are likely to influence the concentration of each compound.

Due to the complexities of hop secondary metabolite composition and the effects of both maturation and environmental stimuli, MAS could be a useful method for breeding hops with improved brewing characteristics; allowing direct selection of hops with improved content and quality of bitter acids and essential oils in the cone. However, deployment of MAS requires a deeper understanding of the complex genetics underlying the synthesis of secondary metabolites that influence bitterness, flavour and aroma of beer. To date, QTL have been identified for a small number of important hop chemical components. In the case of hop essential oils, QTL have been identified for caryophyllene and farnesene [14]; for polyphenols, QTL have been identified for xanthohumol and desmethylxanthohumol [14,18]; and for hop acids, QTL have been identified for α-acid, β-acid, cohumulone (as a percentage of α-acid) and colupulone (as a percentage of β-acid) [14,16]. Five chalcone synthase genes (*vps, chs_*H1, *chs*2, *chs*3 and *chs*4) encoding enzymes directly involved in the biosynthesis of hop acids and polyphenols [64,69-72] have been cloned; these candidate genes have been mapped in one hop population [16]. These studies have barely scratched the surface of the hop secondary metabolite profile, warranting further analysis to identify QTL for secondary metabolites key to beer bittering, flavour and aroma.

In this study, we performed comprehensive QTL analyses, encompassing 50 traits related to three key targets in the genetic improvement of hop: expediting plant sex identification, increasing yield capacity and improving secondary metabolite composition. In order to identify QTL, male and female linkage maps were constructed from two mapping populations using a number of marker systems, including transferable DArT markers developed in this study. In one population we performed QTL analysis on two yield traits and α-acid content, with the goal of identifying environmentally and ontogenetically stable QTL. In the second population we analysed α-acid content and an additional 47 traits related to yield and secondary metabolites, the majority of which have not been previously assessed in hop QTL analyses, in order to identify QTL from single-year data. Both populations were screened for known sex-linked markers and used to search for new ones. Through the analysis of multiple traits over numerous years, this work contributes to our understanding of the genetic basis underlying phenotypic variation in hop, an essential prerequisite for future genetic improvement programs in hop.

## Results

### Marker discovery and linkage analysis

In this study, DArT marker discovery identified 511 new polymorphic markers in hop, from 6,439 DArT clones, resulting in a frequency of polymorphism of 7.9%. A total of 834 DArT markers (511 identified in this study and 323 markers identified in a previous study [73]) were polymorphic in at least one of the two mapping populations and subsequently used for genotyping. The quality of the 834 DArT markers was assessed through several parameters. The average polymorphism information content (PIC) value was 0.36 (SE ± 0.005). Scoring reproducibility, call rate and Q values averaged at 99.8% (SE ± 0.009), 92.2% (SE ± 0.237) and 76.4% (SE ± 0.378), respectively. The New Zealand population was genotyped with an additional 43 microsatellite markers, four RAPD markers, three sequence-tagged site (STS) markers and one marker based on a microsatellite within a candidate chalcone synthase gene (*chs_*H1). The analyses of the Slovenian population included an additional 44 microsatellite markers, 241 AFLP markers and five markers based on microsatellites within candidate chalcone synthase genes that were genotyped in a previous study of the population (*vps, chs_*H1, *chs*2, *chs*3 and *chs*4) [16].

Linkage analysis of the New Zealand maternal ‘Nugget’ population included 337 markers (299 DArT, 34 microsatellite, 2 RAPD, 1 STS, 1 candidate gene) and resulted in a total of 286 markers (264 DArT, 20 microsatellite, 2 RAPD) placed on the map at 80 unique positions (Table 1; Additional file 1). Eleven linkage groups were formed, comprising a total map length of 231.8 cM (Table 1; Additional file 1). Linkage analysis of the New Zealand paternal Slovenian breeding line (S.B.L.) 3/3 population included 189 markers (166 DArT, 17 microsatellite, 3 RAPD, 2 STS, 1 candidate chalcone synthase gene) and resulted in a total of 157 markers (146 DArT, 8 microsatellite, 2 STS, 1 candidate gene) placed on the map at 42 unique positions (Table 1; Additional file 2). Eight linkage groups were formed, comprising a total map length of 243.0 cM (Table 1; Additional file 2). Through comparison between the maternal and paternal linkage maps, and to linkage maps of the Slovenian mapping population (‘Hallertauer Magnum’ × ‘S.B.L. 2/1’) constructed in this study, several homologous linkages were identified (Additional file 3). Where there were markers in common within these homologous linkage groups, the marker order was mostly conserved. There was evidence from homologous linkage groups to show that two of the linkage groups of the maternal ‘Nugget’ map are likely to be from the same chromosome, thus forming a total of ten linkage groups (Additional file 1). These ten linkage groups formed in the maternal ‘Nugget’ map are equal to the haploid number of chromosomes in hop (*n* = 10); however, only eight linkage groups were resolved in the paternal ‘S.B.L. 3/3’ map.

**Table 1:** Comparative features of the maternal and paternal linkage maps of the New Zealand and Slovenian mapping populations

	**New Zealand population**		**Slovenian population**	
	**Nugget**	**S.B.L. 3/3**	**Hallertauer Magnum**	**S.B.L. 2/1**
	**♀**	**♂**	**♀**	**♂**
No. markers on map	286	157	169	121
No. unique positions on map	80	42	106	63
No. linkage groups formed	10/11	8	10 / 14	10 / 11
cM of the genome covered	231.8	243.0	555.8	306.3
Average distance between markers	3.3	7.1	6.1	5.9
Largest interval between markers	36.3	36.1	40.9	32.5
No. markers with segregation distortion	136	127	68	76

Linkage analysis of the Slovenian maternal ‘Hallertauer Magnum’ population included 247 markers (122 DArT, 105 AFLP, 16 SSR, four candidate chalcone synthase genes) and resulted in 169 markers (100 DArT, 52 AFLP, 13 SSR, four candidate chalcone synthase genes) placed on the map at 106 unique positions (Table 1; Additional file 4). Fourteen linkage groups were formed, comprising a total map length of 555.8 cM (Table 1; Additional file 4). Linkage analysis of the Slovenian paternal S.B.L. 2/1 population included 189 markers (84 DArT, 87 AFLP, 18 SSR) and resulted in 121 markers (68 DArT, 38 AFLP, 15 SSR) placed on the map at 63 unique positions (Table 1; Additional file 5). Eleven linkage groups were formed, comprising a total map length of 306.3 cM (Table 1; Additional file 5). Through comparison between the maternal and paternal linkage maps, and to a previously reported map of the family ‘Hallertauer Magnum’ × ‘S.B.L. 2/1’ [16] (Additional file 3), several homologous linkage groups could be identified. Where there were markers in common within these homologous linkage groups, the previously established marker order was mostly conserved. There was evidence from homologous linkage groups to show that several of the linkage groups within both the maternal and paternal maps were likely to be from the same chromosomes, thus forming a total of ten linkage groups in both the maternal and paternal map (Additional files 4 and 5). This is equal to the haploid chromosome number in hop.

The marker derived from the candidate chalcone synthase gene that was included in the linkage analysis of the New Zealand mapping population (*chs*_H1) was polymorphic and mapped to LG 8 of the paternal ‘S.B.L. 3/3’ map (Additional file 2). Of the five markers derived from candidate chalcone synthase genes that were included in the linkage analysis of the Slovenian population, four were polymorphic (*vps, chs_*H1, *chs*2 and *chs*4), and also mapped to LG 8 on the maternal ‘Hallertauer Magnum’ map (Additional file 2), following the same marker order as previously established [16].

Extensive clustering of markers was observed in the linkage maps of both the New Zealand and Slovenian mapping populations (Additional files 1 and 2). All marker types included in linkage analyses exhibited clustering within and between marker types. Before QTL analysis, superfluous markers within each cluster were eliminated to leave only one marker at each locus. In the New Zealand population, a total of 206 and 120 markers were removed from the maternal and paternal linkage maps, respectively; and a total of 63 and 58 markers were removed from the maternal and paternal linkage maps of the Slovenian population. In this study, a significant proportion of markers demonstrated a departure from expected Mendelian segregation ratios (segregation distortion; α < 0.05). Significant segregation distortion was found in all marker types and on all linkage maps constructed (Table 1). Markers with segregation distortion were frequently found close together on the linkage maps, such that the observed marker clusters consisted of markers either with or without segregation distortion. This phenomenon often resulted in entire linkage groups of exclusively distorted or non-distorted markers, or linkage groups divided into these regions (Additional files 1 and 2).

### Phenotypic measurements

Sex was assessed as a binary trait; with 153 female and 25 male plants identified in the New Zealand population, giving a sex ratio of 6.1:1 (female:male). Eighty-seven female and five male plants were recognised in the Slovenian population, giving a sex ratio of 17.4:1 (female:male). All other traits assessed in this study were quantitative (Table 2). Of the three traits assessing hop cone yields, dry cone weight showed the smallest phenotypic variation (SD ± 0.083); followed by cone harvest index (SD ± 0.199); with green cone weight showing an eight-fold difference in variability (SD ± 0.639) compared to dry cone weight (Table 2). The yield of essential oil was also assessed; on average 0.64 ml (SD ± 0.08) of essential oil was obtained from 100 g of dried hop cone tissue (Table 2).

**Table 2:** Phenotypic mean, rage and SD of secondary metabolite and yield traits quantified in the progeny of two hop mapping crosses: (i) Hallertauer Magnum × S.B.L. 2/1, grown in Slovenia; and (ii) Nugget × S.B.L. 3/3, grown in New Zealand

**Chemical group**		**Trait**	**Units**	**Mean**	**Min**	**Max**	**SD**	**Population**	**Measurement years**
hop acid	α-acid	α-acid (LCV measure)	% of dry hop cone weight	8.29	2.75	15.32	2.17	Slovenia	2002-2006
		α-acid	% of dry hop cone weight	5.98	2.03	9.80	1.46	New Zealand	2009
		humulone + adhumulone	% of dry hop cone weight	4.47	1.60	7.87	1.14	New Zealand	2009
		cohumulone	% of dry hop cone weight	1.50	0.42	2.84	0.44	New Zealand	2009
		cohumulone (% of α-acid)	% of α-acid	25.25	17.53	34.71	4.18	New Zealand	2009
	β-acid	β-acid	% of dry hop cone weight	2.17	0.74	4.39	0.65	New Zealand	2009
		lupulone + adlupulone	% of dry hop cone weight	1.10	0.38	2.17	0.33	New Zealand	2009
		colupulone	% of dry hop cone weight	1.07	0.36	2.32	0.35	New Zealand	2009
		colupulone (% of β-acid)	% of β-acid	48.99	41.63	57.62	3.55	New Zealand	2009
	ratio	α-acid:β-acid	ratio of α-acid to β-acid	2.82	1.85	3.86	0.46	New Zealand	2009
essential oil	ester	geranyl acetate	% of total essential oil	0.25	0.00	0.69	0.14	New Zealand	2009
		geranyl isobutyrate	% of total essential oil	0.42	0.00	2.55	0.35	New Zealand	2009
		methyl decanoate	% of total essential oil	0.29	0.00	0.50	0.09	New Zealand	2009
		methyl dec-4-enoate	% of total essential oil	1.24	0.26	3.33	0.55	New Zealand	2009
		methyl-4-methylhex-2-enoate	% of total essential oil	0.37	0.00	1.62	0.27	New Zealand	2009
	ketone	2-undecanone	% of total essential oil	0.33	0.06	0.87	0.19	New Zealand	2009
	ether	humulene diepoxide a	% of total essential oil	0.50	0.00	2.37	0.39	New Zealand	2009
		humulene epoxide I	% of total essential oil	0.29	0.00	1.41	0.21	New Zealand	2009
		humulene epoxide II	% of total essential oil	0.66	0.19	2.84	0.41	New Zealand	2009
		humulene epoxide III	% of total essential oil	0.74	0.12	2.54	0.43	New Zealand	2009
	monoterpene alcohol	geraniol	% of total essential oil	0.78	0.09	2.92	0.39	New Zealand	2009
		limonene-10-ol	% of total essential oil	0.29	0.00	1.94	0.25	New Zealand	2009
		linalool	% of total essential oil	0.43	0.00	1.02	0.21	New Zealand	2009
	sesquiterpene alcohol	caryolan-1-ol	% of total essential oil	0.35	0.00	1.15	0.19	New Zealand	2009
		humulenol II	% of total essential oil	0.06	0.00	0.29	0.08	New Zealand	2009
		humulol	% of total essential oil	0.22	0.00	0.58	0.10	New Zealand	2009
		t-cadinol	% of total essential oil	0.14	0.00	0.40	0.13	New Zealand	2009
	alkane	tetradecane	% of total essential oil	0.10	0.00	0.20	0.05	New Zealand	2009
	monoterpene	β-pinene	% of total essential oil	0.26	0.00	0.69	0.16	New Zealand	2009
		camphene	% of total essential oil	0.05	0.00	0.37	0.07	New Zealand	2009
		limonene	% of total essential oil	0.68	0.00	3.42	0.48	New Zealand	2009
		myrcene	% of total essential oil	28.47	1.13	59.65	0.30	New Zealand	2009
		ρ-cymene	% of total essential oil	0.21	0.00	0.65	13.86	New Zealand	2009
		terpinene	% of total essential oil	0.47	0.00	2.72	0.09	New Zealand	2009
	sesquiterpene	α-capaene	% of total essential oil	0.32	0.00	0.65	0.13	New Zealand	2009
		α-selinene	% of total essential oil	1.21	0.34	2.91	0.52	New Zealand	2009
		β-selinene	% of total essential oil	0.47	0.00	1.33	0.20	New Zealand	2009
		δ-cadinene	% of total essential oil	0.70	0.09	3.24	0.54	New Zealand	2009
		γ-cadinene	% of total essential oil	1.58	0.00	3.73	0.86	New Zealand	2009
		caryophyllene	% of total essential oil	12.37	4.64	22.80	3.90	New Zealand	2009
		caryophyllene oxide	% of total essential oil	0.21	0.00	0.58	0.12	New Zealand	2009
		farnesene	% of total essential oil	7.29	0.06	28.13	7.66	New Zealand	2009
		humulene	% of total essential oil	29.70	9.90	55.92	9.29	New Zealand	2009
		muurolene	% of total essential oil	0.92	0.29	1.74	0.79	New Zealand	2009
	ratio	humulene:caryophyllene	ratio of humulene to caryophyllene	2.50	1.36	3.55	0.66	New Zealand	2009
poly-phenol	poly-phenol	xanthohumol	% of dry hop cone weight	0.24	0.08	0.51	0.46	New Zealand	2009
	secondary metabolites	essential oil content	ml of oil per 100 g of hop cone tissue	0.64	0.17	1.71	0.08	New Zealand	2009
yield	cones	cone harvest index	ratio of cone weight to whole plant weight	0.31	0.11	1.27	0.20	Slovenia	2002-2006
		dry cone weight	kg of dry cones per plant	0.15	0.04	0.40	0.08	Slovenia	2002-2006
		green cone weight	kg green cones per plant	1.54	0.30	3.35	0.64	New Zealand	2009

**Figure 1:**
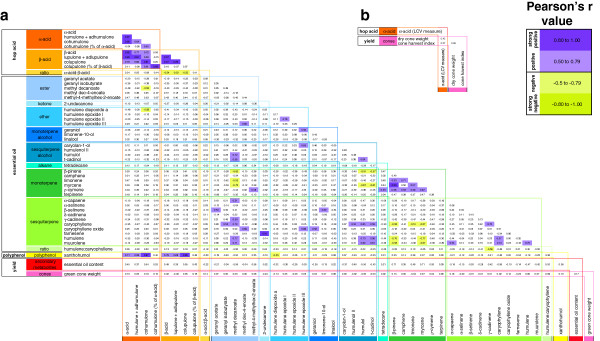
Correlation coefficients (Pearson’s r) for the relationships between secondary metabolite and yield traits examined in hop, in (a) a New Zealand mapping population (n = 47) and (b) a Slovenian mapping population (n = 3).

The secondary metabolite profile of hop was examined in the progeny of a New Zealand mapping cross through a total of 45 traits from all hop secondary metabolite groups (hop acids, essential oils and polyphenols). Quantitatively, the hop acid component of the secondary metabolite profile of the New Zealand mapping population was dominated by α-acid (average 6.0% of dry cone weight), the largest component of which was the humulone + adhumulone fraction (average 4.5% of dry cone weight) (Table 2). The essential oil component of the secondary metabolite profile was dominated by the sesquiterpenes humulene (average 29.7% of total essential oil), caryophyllene (average 12.4% of total essential oil) and farnesene (average 7.3% of total essential oil); and the monoterpene myrcene (average 28.5% of total essential oil) (Table 2). A single polyphenol was assessed, xanthohumol, which comprised an average of 0.2% of the dry cone weight (Table 2). Correlations were evident between a number of the secondary metabolites, both within and between the major structural groups (Figure 1). The strongest correlations were exhibited within the hop acid groups, where the six secondary metabolite traits measured (α-acid, β-acid, humulone + adhumulone, cohumulone, lupulone + adlupulone and colupulone) all shared very strong positive correlations (Pearson’s r > 0.80) (Figure 1). Very strong positive correlations were also observed between several of the other secondary metabolite traits, although many of these correlations did not form cohesive patterns either within or between major chemical groups (Figure 1). The highest phenotypic correlations were between the two hop acids, cohumulone (% of α-acid) and colupulone (% of β-acid) (r = 0.88); the polyphenol and hop acid, xanthohumol and cohumulone (r = 0.87); the polyphenol and hop acid, xanthohumol and colupulone (r = 0.85); the two monoterpenes β-pinene and myrcene (r = 0.96); and the ketone and sesquiterpene, 2-undecanone and farnesene (r = 0.91) (Figure 1). No very strong negative correlations (r < − 0.80) were observed between the secondary metabolites (Figure 1). One secondary metabolite trait, α-acid, was examined in the Slovenian population, measuring an average of 8.3% of dry cone weight. Alpha-acid was not strongly correlated with any other trait measured in the Slovenian population.

### QTL analysis

#### Sex trait

**Figure 2:**
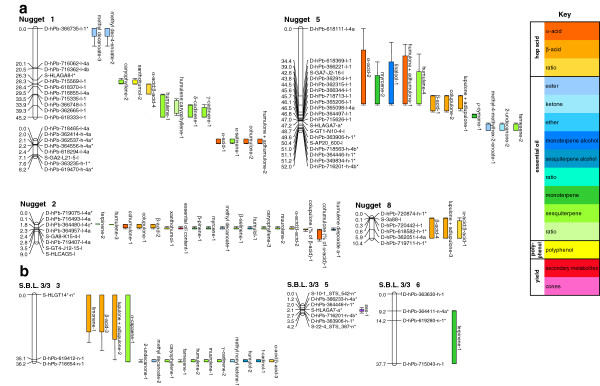
Location of QTL for sex, yield and secondary metabolite traits on (a) maternal and (b) paternal linkage groups of hop from the New Zealand mapping population.

**Figure 3:**
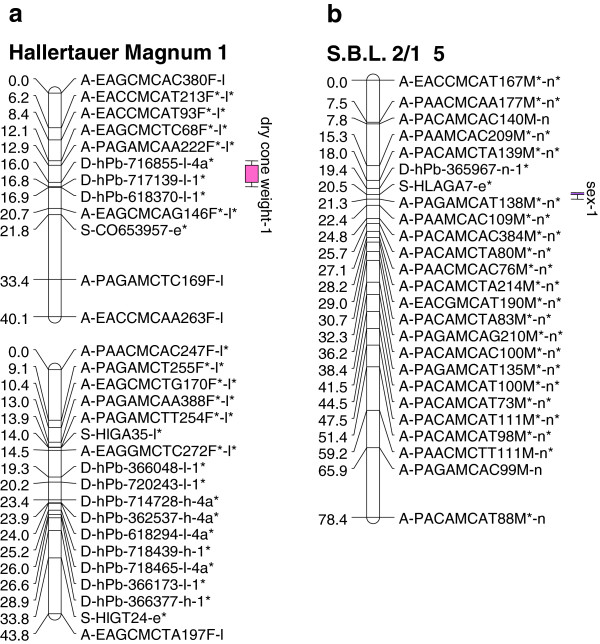
Location of QTL for sex and yield traits on (a) maternal and (b) paternal linkage groups of hop from the Slovenian mapping population.

One sex-linked marker was detected in this study, identified for the first time in the New Zealand population and confirming its previous identification in the Slovenian population (Table 3; Figures 2 and 3). In both cases, the microsatellite marker HLAGA7 segregated from the male parent and showed complete linkage to the male character. While highly significant in both populations, differences in the level of significance were apparent, with a higher significance observed in the Slovenian population (LOD 1441; Table 3b) than in the New Zealand population (LOD 14; Table 3a).

**Table 3:** Quantitative trait loci identified for sex, yield and cone chemistry traits in hop, in (a) a New Zealand mapping population and (b) a Slovenian mapping population

**Chemical group**		**Trait**	**QTL**	**Linkage group**	**Seg. **^***a***^	**Adjacent marker**	**Position (cM)**	**LOD **^***b***^	**% exp **^***c***^	**Additive **^***d***^	**Kruskal-Wallis**
**a.**											
hop acid	α-acid	α-acid	α-acid-1	Nugget 1	F	D-hPb-718465-l-4af	0.0	2.91	8.6	0.43	P < 0.0005
			α-acid-2	Nugget 5	F	D-hPb-618369-l-1f	34.4	2.62	7.1	0.40	P < 0.0005
		humulone + adhumulone	humulone + adhumulone-1	Nugget 5	F	D-hPb-618369-l-1f	34.4	4.26	11.3	0.39	P < 0.0001
			humulone + adhumulone-3	Nugget 1	F	D-hPb-718465-l-4af	0.0	2.58	6.7	0.29	P < 0.001
		cohumulone	cohumulone-1	Nugget 2	F	D-hPb-364480-l-4c*f	1.8	8.14	21.9	−0.21	P < 0.0001
			cohumulone-2	Nugget 1	F	D-hPb-718465-l-4af	0.0	3.29	8.1	0.12	P < 0.0005
		cohumulone (% of α-acid)	cohumulone (% of α-acid)-1	Nugget 2	F	S-GT4-J12-15-lf	3.5	24.78	43	−2.78	P < 0.0001
	β-acid	β-acid	β-acid −1	Nugget 5	F	D-hPb-366221-l-1f	39.0	5.01	11.9	0.23	P < 0.0001
			β-acid −2	Nugget 2	F	D-hPb-364480-l-4c*f	1.8	5.01	11.9	−0.23	P < 0.0005
			β-acid −3	S.B.L. 3/3 3	M	S-HLGT14*-n*m	0.0	4.39	10.3	−0.28	P < 0.05
			β-acid-4	Nugget 8	F	D-hPb-362051-l-4af	5.9	2.37	5.4	0.15	P < 0.05
		lupulone + adlupulone	lupulone + adlupulone-1	Nugget 5	F	D-hPb-366221-l-1f	39.0	5.72	14.7	0.13	P < 0.0001
			lupulone + adlupulone-2	S.B.L. 3/3 3	M	S-HLGT14*-n*m	0.0	3.70	9.2	−0.13	P < 0.005
			lupulone + adlupulone-3	Nugget 8	F	D-hPb-362051-l-4af	5.9	3.17	7.8	0.09	P < 0.005
		colupulone	colupulone-1	Nugget 2	F	D-hPb-364480-l-4c*f	1.8	8.40	21.8	−0.16	P < 0.0001
			colupulone-2	Nugget 5	F	D-hPb-366221-l-1f	39.0	3.55	8.5	0.10	P < 0.0005
		colupulone (% of β-acid)	colupulone (% of β-acid)-1	Nugget 2	F	D-hPb-719407-l-4af	2.8	20.54	44.4	−2.4	P < 0.0001
	ratio	α-acid:β-acid	α-acid:β-acid-1	Nugget 8	F	D-hPb-362051-l-4af	5.9	4.44	10.5	−0.15	P < 0.0005
			α-acid:β-acid-2	Nugget 2	F	D-hPb-364957-l-4af	2.3	4.40	10.4	0.15	P < 0.0005
			α-acid:β-acid-3	S.B.L. 3/3 3	M	D-hPb-716654-n-1 m	36.2	3.63	8.5	0.13	P < 0.001
			α-acid:β-acid-4	Nugget 1	F	D-hPb-716855-l-4af	29.5	2.65	6.1	0.11	P < 0.01
essential oil	ester	methyl decanoate	methyl decanoate-1	Nugget 2	F	D-hPb-364957-l-4af	2.3	6.91	17.2	0.04	P < 0.0001
			methyl decanoate-2	S.B.L. 3/3 3	M	D-hPb-716654-n-1 m	36.2	3.70	8.7	−0.03	P < 0.0001
			methyl decanoate-3	Nugget 1	F	D-hPb-366735-l-1*f	0.0	2.45	5.6	−0.03	P < 0.001
		methyl dec-4-enoate	methyl dec-4-enoate-2	Nugget 1	F	D-hPb-366735-l-1*f	0.0	3.81	12.3	0.21	P < 0.0001
		methyl-4-methylhex-2-enoate	methyl-4-methylhex-2-enoate-2	Nugget 5	F	S-AP20_600-lf	50.4	2.36	6.1	0.07	P < 0.01
	ketone	2-undecanone	2-undecanone-1	S.B.L. 3/3 3	M	D-hPb-716654-n-1 m	36.2	25.1	53.8	0.14	P < 0.0001
			2-undecanone-2	Nugget 5	F	S-AP20_600-lf	50.4	4.62	6.8	−0.05	P < 0.01
	ether	humulene diepoxide a	humulene diepoxide a-1	Nugget 2	F	S-GA8-K15-4-lf	2.4	2.37	7.8	0.11	P < 0.05
	monoterpene alcohol	linalool	linalool-1	Nugget 5	F	D-hPb-618369-l-1f	34.4	2.68	8.8	0.06	P < 0.005
	sesquiterpene alcohol	humulol	humulol-1	Nugget 2	F	D-hPb-364957-l-4af	2.3	3.81	11.2	0.04	P < 0.0001
			humulol-2	S.B.L. 3/3 3	M	D-hPb-716654-n-1 m	36.2	2.80	8.1	−0.03	P < 0.0005
		t-cadinol	t-cadinol-1	S.B.L. 3/3 3	M	D-hPb-716654-n-1 m	36.2	3.34	10.1	−0.03	P < 0.0005
	monoterpene	β-pinene	β-pinene-1	Nugget 2	F	D-hPb-364957-l-4af	2.3	5.42	16.1	−0.07	P < 0.0001
		limonene	limonene-1	S.B.L. 3/3 3	M	S-HLGT14*-n*	0.0	2.33	7.7	−0.17	P < 0.001
		myrcene	myrcene-1	Nugget 2	F	D-hPb-364957-l-4af	2.3	5.54	16	−5.61	P < 0.0001
			myrcene-2	Nugget 5	F	D-hPb-618369-l-1f	34.4	3.56	9.9	4.43	P < 0.005
		ρ-cymene	ρ-cymene-1	Nugget 5	F	D-hPb-362315-l-1f	44.5	2.84	9.3	0.04	P < 0.0005
		terpinene	terpinene-1	S.B.L. 3/3 6	M	D-hPb-619280-n-1*m	14.2	3.19	9.3	0.14	P < 0.0001
			terpinene-2	Nugget 2	F	D-hPb-719075-l-4a*f	0.0	2.73	7.9	−0.13	P < 0.0001
	sesquiterpene	α-capaene	α-capaene-1	S.B.L. 3/3 3	M	D-hPb-619412-n-1 m	35.1	3.52	11.4	−0.05	P < 0.0005
		α-selinene	α-selinene-1	Nugget 1	F	D-hPb-718465-l-4af	0.0	4.01	12.9	−0.19	P < 0.0001
		β-selinene	β-selinene-1	Nugget 2	F	D-hPb-364957-l-4af	2.3	3.88	11.5	0.07	P < 0.0001
		δ-cadinene	δ-cadinene-1	Nugget 1	F	D-hPb-618333-l-1f	45.2	6.05	18.8	0.23	P < 0.0001
		γ-cadinene	γ-cadinene-1	Nugget 1	F	D-hPb-618333-l-1f	45.2	7.87	22.6	0.41	P < 0.0001
			γ-cadinene-2	S.B.L. 3/3 3	M	D-hPb-716654-n-1 m	36.2	2.67	7	−0.23	P < 0.05
		caryophyllene	caryophyllene-1	S.B.L. 3/3 3	M	D-hPb-716654-n-1 m	36.2	6.23	13.7	−1.45	P < 0.0001
			caryophyllene-2	Nugget 1	F	D-hPb-715569-l-1	28.3	5.45	12.6	1.39	P < 0.005
			caryophyllene-3	Nugget 2	F	D-hPb-364957-l-4af	2.3	5.10	11	1.32	P < 0.0001
		farnesene	farnesene-1	S.B.L. 3/3 3	M	D-hPb-716654-n-1 m	36.2	39.78	71.3	6.45	P < 0.0001
			farnesene-2	Nugget 5	F	S-AP20_600-lf	50.4	4.62	4.2	−1.62	P < 0.05
		humulene	humulene-1	Nugget 1	F	D-hPb-362665-l-1f	39.3	7.15	15.3	−3.67	P < 0.0001
			humulene-2	S.B.L. 3/3 3	M	D-hPb-716654-n-1 m	36.2	6.42	13.8	−3.45	P < 0.0001
			humulene-3	Nugget 2	F	D-hPb-719075-l-4a*f	0.0	3.61	7.3	2.55	P < 0.01
			humulene-4	Nugget 5	F	D-hPb-618369-l-1f	34.4	2.66	5.3	−2.22	P < 0.005
		muurolene	muurolene-1	S.B.L. 3/3 3	M	D-hPb-716654-n-1 m	36.2	8.83	22.6	−0.15	P < 0.0001
			muurolene-2	Nugget 2	F	D-hPb-364957-l-4af	2.3	5.17	12.4	0.11	P < 0.0001
	ratio	humulene:caryophyllene	humulene:caryophyllene-1	Nugget 1	F	D-hPb-362665-l-1f	39.3	38.44	66.9	−0.54	P < 0.0001
poly-phenol	poly-phenol	xanthohumol	xanthohumol-1	Nugget 2	F	D-hPb-364957-l-4af	2.3	11.5	29.5	−0.05	P < 0.0001
			xanthohumol-2	Nugget 1	F	D-hPb-715569-l-1f	28.3	2.46	5.4	−0.02	P < 0.01
yield	secondary metabolites	essential oil content	essential oil content-1	Nugget 2	F	D-hPb-364957-l-4af	2.3	6.66	20.1	−0.16	P < 0.0001
sex	sex	sex	sex-1	S.B.L. 3/3 5	M	S-HLAGA7-a*m	2.1	13.84	22.6	−0.28	P < 0.0001
**b.**											
yield	cone	dry cone weight	dry cone weight-1	Hallertauer Magnum 1	F	D-hPb-716855-l-4a*f	16.0	7.49	35.0	0.05	P < 0.0001
sex	sex	sex	sex-1	S.B.L. 2/1 5	M	S-HLAGA7-e*	20.5	1441.23	81	0.50	P < 0.0001

*a Seg.* indicates the segregation of the QTL from either the maternal (F) or paternal (M) parent.

*b LOD* indicates the peak LOD score for the QTL at the genome-wide significance level.

*c % exp.* indicates the percentage of the phenotypic variation of the trait explained by the QTL.

*d Additive* indicates the estimated additive effect of the allele (i.e. (mean of the distribution of the quantitative trait associated with the female genotype – mean of the distribution of the quantitative trait associated with the male genotype)/2.

#### Yield traits

Of the four yield traits assessed in this study, QTL were identified for two: one QTL for essential oil content (Table 3a; Figure 2a) and one QTL for dry cone weight (Table 3b; Figure 3b). Both QTL segregated from the female parent, explaining 20.1% and 35.0% of the phenotypic variation, respectively (Table 3). QTL were not identified for cone harvest index or green cone weight.

#### Secondary metabolite traits

A total of 60 putative QTL were identified for hop secondary metabolite traits, above the genome-wide significance level (α < 0.05) (Table 3a). For 33 of the 45 assessed secondary metabolite traits, between one and four QTL were identified, each explaining an estimated 4.2% to 71.3% of the phenotypic variance (Table 3a). QTL were not identified for 12 essential oil components (camphene, caryolan-1-ol, caryophyllene oxide, geraniol, geranyl acetate, geranyl isobutyrate, humulene epoxide I, humulene epoxide II, humulene epoxide III, humulenol II, limonene-10-ol, tetradecanone). QTL were also not identified for α-acid in the Slovenian population.

The 60 putative QTL identified for hop secondary metabolite traits mapped to 13 discreet regions (defined as having QTL peaks separated by more than 5 cM) on six linkage groups (Table 4; Figure 2). Five of the QTL regions were unique to specific traits, these being humulene (QTL region 3) on ‘Nugget’ linkage group (LG) 1 (39.3 cM); cadinene (both δ and γ isoforms) (QTL region 4) on ‘Nugget’ LG1 (45.2 cM); terpinene (QTL region 14) on ‘S.B.L. 3/3’ LG6 (14.2 cM); ρ-cymene (QTL region 8) on ‘Nugget’ LG5 (44.5 cM); and lupulone + adlupulone (QTL region 10) on ‘Nugget’ LG7 (5.9 cM) (Table 4; Figure 2). QTL region 10, affecting lupulone + adlupulone, was also found to affect, by extension, the traits β-acid and α-acid:β-acid (Figure 2b), as lupulone + adlupulone is equivalent to β-acid. QTL region 3, affecting humulene was found to affect, by extension, humulene:caryophyllene (Figure 2a), showing that the humulene:caryophyllene ratio is biased towards humulene. The remaining eight QTL regions affected multiple traits (Table 4; Figure 2). Two of these (QTL regions 2 and 6) were found to influence compounds from all three groups of secondary metabolites (hop acids, essential oils and polyphenols), affecting three and 18 different components, respectively (Table 4; Figure 2). The other six QTL regions influenced compounds from one or two different secondary metabolite classes: (i) QTL region 5 influenced four traits, three α-acid compounds and a sesquiterpene (relatively isolated in the biosynthetic pathway from the other sesquiterpenes included in this study); (ii) QTL region 7 influenced eight traits from the hop acid and essential oil groups; (iii) QTL region 9 influenced three traits: an ester, a ketone and a sesquiterpene (isolated in the biosynthetic pathway from the other secondary metabolites included in this study); (iv) QTL region 11 influenced three traits: two from the β-acid group and one sesquiterpene; (v) QTL region 12 influenced 11 traits from the hop acid and essential oil groups (Table 4; Figure 2); and (vi) QTL region 1 influenced two traits, both esters (Table 4; Figure 2). Each of the QTL regions identified in this study were sex-specific; 10 of the 13 QTL regions segregated from the female parent ‘Nugget’ (Table 4), a significant bias (χ^2^_1_ = 3.8, P < 0.05).

**Table 4:** Discrete QTL, both specific and pleiotropic/linked, identified in hop; and the sex, yield and cone chemistry traits affected by each locus

**QTL region**	**Specificity**	**Linkage group**	**No. traits**	**Traits**
QTL region 1	pleiotropic	Nugget 1	2	methyl decanoate; methyl dec-4-enoate
QTL region 2	pleiotropic	Nugget 1 & Hallertauer Magnum 1	4	α-acid:β-acid; caryophyllene; xanthohumol; dry cone weight
QTL region 3	specific	Nugget 1	2	humulene; humulene:caryophyllene
QTL region 4	specific	Nugget 1	2	δ-cadinene; γ-cadinene
QTL region 5	pleiotropic	Nugget 1	4	α-acid; humulone + adhumulone; cohumulone; α-selinene
QTL region 6	pleiotropic	Nugget 2	18	cohumulone; cohumulone (% of α-acid); β-acid; colupulone; colupulone (% of β-acid); α-acid:β-acid; methyl decanoate; humulene diepoxide a; humulol;, β-pinene; myrcene; terpinene; β-selinene; caryophyllene; humulene; muurolene; xanthohumol; essential oil content
QTL region 7	pleiotropic	Nugget 5	8	α-acid; humulone + adhumulone; β-acid; lupulone + adlupulone; colupulone; linalool; myrcene; humulene
QTL region 8	specific	Nugget 5	1	ρ-cymene
QTL region 9	pleiotropic	Nugget 5	3	methyl-4-methylhex-2-enoate; 2-undecanone; farnesene
QTL region 10	specific	Nugget 7	3	β-acid; lupulone + adlupulone; α-acid:β-acid
QTL region 11	pleiotropic	S.B.L. 3/3 3	3	β-acid; lupulone + adlupulone; limonene
QTL region 12	pleiotropic	S.B.L. 3/3 3	11	α-acid:β-acid; methyl decanoate; 2-undecanone; humulol; t-cadinol; α-capaene; γ-cadinene; caryophyllene; farnesene; humulene; muurolene
QTL region 13	specific	S.B.L. 3/3 5 & S.B.L. 2/1 5	1	sex
QTL region 14	specific	S.B.L. 3/3 6	1	terpinene

Comparisons between QTL identified in the New Zealand and Slovenian populations were made using markers found in common between linkage maps constructed from the two populations. The QTL for dry cone weight that was identified in the Slovenian population (Table 3; Figure 3a) co-located with QTL region 2, on ‘Nugget’ LG1 (29.5 cM) of the New Zealand population, influencing the three traits α-acid:β-acid, caryophyllene and xanthohumol (Table 4). Of the five markers based on candidate chalcone synthase genes (*vps, chs_*H1, *chs*2, *chs*3 and *chs*4) included in linkage analysis of the Slovenian population in this study, four were mapped, in the maternal ‘Hallertauer Magnum’ LG8 of the Slovenian population. Although these genes encode enzymes involved in the biosynthesis of hop acids and poloyphenols, no QTL for α-acid was identified in the Slovenian population, associated with these chalcone synthase genes or on any other marker. Two QTL were, however, identified for α-acid in the New Zealand mapping population (explaining 8.6% and 7.1% of the phenotypic variation, respectively), as well as an additional 19 QTL for other hop acid traits and two QTL for xanthohumol (Table 3). None of the QTL identified were associated with the marker based on a candidate chalcone synthase gene (*chs*_H1) that mapped to ‘S.B.L. 3/3’ LG8 of the New Zealand population.

## Discussion

### Marker discovery and linkage analysis

Although linkage maps derived from four hop mapping populations have been published [14-17], a highly saturated linkage map is still to be constructed for this species. The high resolution of such a map is an essential component for the identification of accurate and reproducible QTL, particularly those with small effects. The goal of this study was to construct linkage maps for a new mapping population from New Zealand and to build upon a pre-existing linkage map from a Slovenian mapping population [16], through the addition of transferable DArT markers. The 511 new DArT markers identified in this study were found to be of a similar high quality (in terms of PIC, reproducibility and call rate) to those identified in a previous hop study [73] and in other plant species [74-78]. The use of these new DArT markers in linkage analysis of the Slovenian population, along with DArT, AFLP and microsatellite markers previously identified [16,73,79], increased the number and density of markers, allowed the clear identification of ten linkage groups (corresponding to the haploid number of chromosomes in hop) and increased the transferability of the maps between mapping populations. Our study used a conservative approach; accepting only markers within designated parameters (see Methods below). This method, in combination with factors such as the amalgamation of some of the smaller groups and the exclusion of terminal markers, resulted in shorter map lengths compared with Cerenak et al. [16]. Where there were markers in common between the linkage maps of the Slovenian population constructed in this study and previous maps, marker order was mostly conserved and homologous linkage groups were identified (Additional file 3). Linkage group homology and marker order was also consistent between linkage maps constructed using the Slovenian population and the New Zealand population, and between maternal and paternal linkage maps of both populations (Additional file 3). While ten linkage groups were identified in the maternal linkage map of the New Zealand population (Additional file 1), only eight were resolved in the paternal map (Additional file 2). The maternal linkage map also contained nearly double the number of markers of the paternal linkage map. These factors suggest that further addition of markers is required to achieve genome coverage in the paternal linkage map of the New Zealand population.

In this study, a large number of markers demonstrated significant departure from expected Mendelian ratios (Table 1; Additional files 1 and 2). Significant clustering of the markers was also observed (Additional files 1 and 2). These phenomena have been previously identified in other species and were attributed to biological factors, rather than experimental limitations [80-88]. Several factors indicate that this was also the case in this study. Both clustering of markers and segregation distortion (which has been identified in hop previously [15,16]) was not limited to one marker type, but was evident in all marker types used, suggesting that they are not artefacts of genotyping error. Marker clusters were associated with regions of segregation distortion, such that they were composed of either all distorted markers or all non-distorted markers. Clustering of markers is typically symptomatic of saturation of markers on the linkage map [89], yet marker saturation in this study is unlikely, for several reasons. Intervals of greater than 10 cM exist (Table 1); and in one of the four linkage maps the number of linkage groups identified was fewer than the number of hop chromosomes, while in the other three linkage maps several of the hop chromosomes were split into two or more linkage groups because of insufficient linkage. These factors indicate that the linkage maps do not contain the maximal density of markers and suggest that additional markers are required if smaller sub-groups are to coalesce into a single linkage group. Also, marker saturation in clustered groups may indicate low levels of recombination in hop, for which there is no reported evidence. All of these factors suggest that marker clustering, as well as the segregation distortion of markers, shorter map length and lower marker density of the maps constructed in this study have a biological basis for which further investigation is required.

The linkage maps constructed in this study provide a valuable resource for QTL analyses in hop. The large number of QTL identified in this study (Table 3) provide an excellent starting point to begin to understand the complex genetic architecture underlying variation in hop secondary metabolite composition, which is critical to the ultimate use of hop to provide bitterness, flavour and aroma in beer. Comprising a large number of transferable markers, mapped with a conservative methodology, these linkage maps will provide a basis for further comparative mapping, facilitating the identification and validation of QTL that may ultimately be applied to successful application of molecular methods in selection programs.

### QTL analysis

#### Sex trait

In this study, the microsatellite marker HLAGA7 was linked to the sex phenotype (Table 3; Figures 2 and 3). The association of this marker with sex has been previously reported in two mapping populations grown in Slovenia [22], one of which was the same as the mapping population used in this study. In this study the segregation of this sex-linked marker was detected for the first time in a New Zealand mapping population (Table 3; Figure 2), extending the utility of this marker. In both the New Zealand and Slovenian populations this sex-linked marker segregated from the male parent and showed complete linkage to the male sex phenotype. This is consistent with the specificity of the Y chromosome to the male sex, male plants with the heteromorphic XY configuration and female plants with homomorphic XX [21,29-31]. In both populations the significance of association of the HLAGA7 marker with sex was very high (Table 3); however, a greater level of significance was identified in the Slovenian population. The greater significance in the Slovenian population is likely to reflect differences such as greater map length and number of markers on the linkage group of the Slovenian population (Table 1; Figures 2b and 3b) as well as the smaller size of the Slovenian population, since inflation of QTL effects increases with decreasing population size [90]. Sex-linked molecular markers have been identified in hop previously [19-23]; however, the HLAGA7 marker is the most definitive sex-linked marker identified in hop to date, having now been verified in multiple populations. This study has confirmed the potential for the HLAGA7 marker to be used for routine screening, allowing for the rapid identification of sex in hop breeding programs in diverse environments and populations. Further studies are required, however, to understand the influence of autosomal regions on gender differentiation, as no sex-linked markers were identified on autosomes in this study. This may be due to the existence of numerous regions, each with small effects of too low significance to be detected by this QTL analysis, or the autosomal regions may not contain polymorphism linked to gender differentiation.

#### Yield traits

Three traits related to hop cone yield were examined in this study. Of these, dry cone weight was the only trait for which a QTL was identified. One QTL, stable over the five year period, was detected, segregating from the female parent of the Slovenian population, explaining 35% of the phenotypic variation (Table 3; Figure 3). QTL were not identified for either green cone weight (New Zealand population) or cone harvest index (Slovenian population). The potential reasons for the failure to detect QTL differ between these two yield metrics. Quantification of green cone weight is a method used for the rapid experimental assessment of hop yield. The lack of detectable QTL influencing this trait may be due to the variable moisture content of green cones (which typically contain ~75 – 80% moisture) compared to dry cones (which contain ~8 – 10% moisture). Moisture content of harvested commercial product varies in other species [91-93] and where this is the case, most suggest that it should be corrected for. The lack of QTL for this trait suggest that caution should be applied to using green cone weight as a metric for informing yield.

Harvest index is likewise commonly used to evaluate crop yields and a number of QTL have been identified in various crop species [94-96]. Although heritability of cone harvest index has not been directly examined in hop, the heritability of another hop yield trait has been found to be high [38]. The high phenotypic variability for harvest index (Table 2) suggests that there should be potentially enough power to detect QTL in this study. However, despite the high variability and potentially high heritability, no QTL, stable over the five year period, were detected for cone harvest index. This suggests that, unlike dry cone weight for which a strong QTL was detected, harvest index is influenced by multiple loci, each with small effects, which individually did not have high enough significance to be detected by this QTL analysis. Further investigations are required to elucidate the heritability and genetic basis to variation in this commercially important trait.

QTL for cone yields have been identified in a previous study in the Slovenian population [16], for both dry cone weight and cone harvest index. That QTL analysis, designed to maximally exploit QTL potential, was based on phenotypic measurements of single years (five years examined in total) and identified more than 30 QTL across the years. These QTL were found to be highly variable across the different years, probably due to seasonal variation. The present study had the goal of identifying QTL with significant effects detectable over the entire five-year experimental period. Using data averaged over five years, only a single QTL was identified for dry cone weight that was stable across the five-year experimental period (Table 3; Figure 3). This QTL is adjacent to a marker (A-PAGAMCAA222F*l*) identified as a putative QTL for dry cone weight in a previous study of the Slovenian population [16]. This QTL, based on five-year average data, is less likely to be affected by environmental conditions or horticultural practice (reflected in annual variation) than other putative QTL identified in the previous study [16]. This QTL is an excellent candidate for MAS and warrants further investigation outside of the Slovenian growing region.

Hop essential oils are thought to be the primary contributing factors influencing the flavour and aroma of beer and as such, total essential oil content is an important yield trait. Particular essential oil profiles have historically been targeted in the genetic improvement of hop [97,98]. While it has long been understood that hop essential oil profiles have a genetic basis [99-102], the genetic control of total essential oil content has not been previously established in hop. QTL have been previously identified for only two individual essential oil components, caryophyllene and farnesene [14], but not for total essential oil content. This study is the first to report an underlying genetic basis to variation in the accumulation of essential oils in the hop cone, via the identification of a putative QTL for essential oil content (Table 3; Figure 2). The QTL identified segregated from the female parent of the New Zealand population, explaining a sizeable proportion (20.1%) of the phenotypic variation. The loci underlying variation at this QTL has great potential for hop MAS, in situations where particular levels of essential oil are the target of genetic improvement efforts. Validation outside the pedigree and experimental environment of New Zealand could provide the scope for definitive and heritable increases in yield of total essential oils.

#### Secondary metabolite traits

Although specific secondary metabolites constitute the commercially important hop commodity, our understanding of the genetic basis underlying their variation is in its infancy, with QTL identified for only eight traits related to the control of secondary metabolite content. In this study, we performed an extensive QTL analysis of hop secondary metabolites, investigating 45 hop secondary metabolite traits, 33 of which were found to have a significant genetic basis to their phenotypic variation. A total of 60 putative QTL were identified (Table 3; Figure 2). Between one and four QTL were identified for each of the 33 traits, varying in both their significance (LOD scores ranging between 2.3 and 39.8) and the proportion of phenotypic variance of the trait explained (average 14.9% ± 13.6 SD), suggesting that the composition and concentration of secondary metabolites in hop is influenced by both Mendelian and quantitative inheritance. This is consistent with the genetic studies of secondary metabolites in other genera, such as *Mentha, Thymus* and *Eucalyptus*[103,104]. For example, the occurrence of a single highly significant QTL for individual compounds, such as cohumulone (expressed as percentage of α-acid) and colupulone (expressed as percentage of β-acid) (Table 3; Figure 2a), may be indicative of the influence of major loci with Mendelian inheritance; whereas a greater number of QTL of lesser significance were detected for other compounds, such as humulene, which is consistent with quantitative control (Table 3; Figure 2). No QTL were detected for 12 of the secondary metabolite traits. These secondary metabolite traits all contributed a very low percentage of the secondary metabolite profile and as such may have been subject to inaccuracies in quantification.

Two QTL were identified for α-acid content (New Zealand population) in this study, explaining 8.6% and 7.1%, respectively (Table 3; Figure 2). Although α-acid was also examined in the Slovenian population in this study, we were unable to detect these QTL for α-acid. There may be several reasons for this, including a lack of polymorphism in this population, variable loci effects in different genetic backgrounds (i.e. epistasis), instability of the QTL over varying ontogenetic stages or seasonal conditions, or confounding environmental influences. Evidence of environmental influence on the accumulation of α-acid in hop glandular trichomes has been found in a previous study of the Slovenian population [16], where QTL analysis based on phenotypic measurements of five single years identified 13 QTL for α-acid, but none of these QTL were identified in more than three years, possibly due to seasonal variation. This study re-examined QTL for α-acid in the Slovenian population, conducting QTL analysis on phenotypic data averaged over the five years, with the aim of identifying ontogenetically stable QTL. However, such a QTL was not identified. These results highlight how different ontogenetic stages and seasonal environmental conditions influence the identification of reliable and reproducible QTL and reinforce the requirement for further validation in alternate populations and different environmental conditions to improve our understanding of the genetic basis to variation in this important agronomic trait.

The QTL identified for α-acid in the New Zealand population in this study, as well as the QTL identified for other hop acids and polyphenols, may correspond to regulatory factors rather than genes encoding biosynthetic enzymes. One marker based on a candidate chalcone synthase gene (*chs_*H1) encoding an enzyme involved in the biosynthesis of hop acids was mapped in the New Zealand population in this study, but none of the QTL identified were associated with this gene. Our findings were consistent with those of Cerenak et al. [16], who identified QTL for α-acid, but not associated with chalcone synthase genes. This observation may support the conclusions of Matoušek et al. [69,105], that variation in regulatory factors rather than chalcone synthase genes may have a greater effect on variation in hop acids and polyphenols.

This study identified a gender bias in the inheritance of hop secondary metabolite phenotypes. A total of 13 QTL regions were identified in this study, influencing the 33 secondary metabolite traits. Each of these QTL regions were sex-specific, with 10 QTL regions associated with the female parent and three associated with the male parent (Table 4; Figure 2). This significant partiality towards inheritance from the female parent may be due to several reasons. Firstly, inheritance of the maternal phenotype may be due to dominance of the female parent at these loci; for each of the secondary metabolite traits, segregation may have occurred in the female parent while the corresponding locus in the male parent was homozygous recessive. Secondly, the bias towards maternal inheritance of secondary metabolite traits may be due to epigenetic effects, where inheritable modifications to the activation of genes have occurred to promote the natural selective advantage of a female parent with a favourable secondary metabolite profile; the maternal control over the secondary metabolite profile is maintained in the offspring, passing on the advantage [106-108]. Thirdly, the bias towards inheritance from the female parent may be due to artificial selection, where hop breeders have shown selection bias towards the commercially-relevant female plants. Artificial selection has not, however, been conducted as extensively in the male parents, which often perform as unknown pollinators (with open pollination) in the traditional crossing process [109]). Further research is required to understand the underlying genetic basis for this gender bias in inheritance of secondary metabolite variation.

Co-location of many of the putative QTL identified for secondary metabolites was a striking feature of this study. Thirteen distinct QTL regions were detected, across six linkage groups in the New Zealand population (Table 4). Of these 13 QTL regions, five displayed specificity for individual compounds (Table 4). As hop secondary metabolites are derived from pathways of terpene metabolism and involve common precursors [56,64-68,110], the specificity of the QTL identified for single compounds suggests that these QTL may affect genes, transcription factors or enzymes involved in later stages of biosynthesis and modification of these compounds. Specific QTL for compounds arising from the same biosynthetic pathway have been identified amongst co-locating QTL in several other genetic analyses of secondary metabolites [103,111]. The remaining eight QTL regions detected were found to affect multiple traits (Table 4). Although several strong correlations existed between many of the secondary metabolites in these seven QTL regions (Figure 1), in most cases there were no clear patterns amongst these correlations to coincide with the co-locating QTL or functional grouping of secondary metabolites (Additional file 6). Two of these QTL regions influenced compounds from all three groups of secondary metabolites (hop acids, essential oils and polyphenols), affecting three and 18 individual compounds, respectively (Table 4; Figure 2). The other four QTL regions had a less extensive influence, affecting two to eleven traits and only some of the secondary metabolite groups at one time (Table 4; Figure 2). Through comparisons between the New Zealand and Slovenian linkage maps, the QTL for dry cone weight identified in the Slovenian population was matched to one of the QTL regions on the New Zealand linkage map (QTL region 2 influencing the traits α-acid:β-acid, caryophyllene and xanthohumol; Table 4). Further research is required to elucidate the genetic basis of variation in these traits and the relationships between them.

There may be an underlying genetic basis for the co-location of QTL observed in this study, reflecting pleiotropic effects of single loci influencing multiple secondary metabolite compounds. Pleiotropy is consistent with the conclusion that all of the secondary metabolites of hop lupulin glands are derived from common precursors and pathways of terpene biosynthesis [56,64-68,110,112]. Alternatively, the co-location of these QTL may be due to linkage between the loci associated with the secondary metabolite traits. Loci influencing secondary metabolites often exist in gene families; secondary metabolite diversity is thought to have arisen by gene duplications and consequently, the genes responsible for significant effects on variation in secondary metabolites are likely to be located very close together on the genome [113,114]. Duplication events in secondary metabolite genes, resulting in genetic linkage, have been found in a diversity of species, including *Vitis vinifera*[115], *Arabidopsis thaliana*[116], *Avena sativa*[117] and also hop [72,118]. Therefore, the co-location of QTL identified in this study is likely to reflect the influence of both pleiotropic and linked loci, consistent with the findings of genetic studies of secondary metabolites in other taxa [103,111,119]. The detection of pleiotropy/linkage on the scale determined in this study would not have been possible without the simultaneous examination of an extensive number of traits. Characterising the polymorphism and effects of pleiotropic/linked loci in diverse lineages of hop will be essential for effective application of markers linked to QTL in MAS.

The occurrence of pleiotropic or linked loci in the genetic control of secondary metabolites may have played an important ecological and evolutionary role in hop. The global hop population has been found to encompass limited levels of genetic diversity [73,120-123]. Prior to artificial selection of hop, the existence of pleiotropic or linked loci may have provided an adaptive strategy, assisting in the selective adaptation of hop, as a defensive mechanism against pathogens, for example. Mutations in single genes from pleiotropic loci could affect the biosynthesis and profile of a large number of secondary metabolites, enabling a rapid diversification of secondary metabolite profiles and a broader defence response, compared to changes to single secondary metabolites by compound-specific genes. Alternatively, the occurrence of pleiotropic or linked loci may also be an artefact of selection during and since hop domestication. Artificial selection of hops for particular brewing characteristics and distinct chemical profiles may have resulted in the inheritable linkage of particular combinations of secondary metabolites. The effect of artificial selection on the genetic linkage of a number of different traits has been reported previously in a range of species [124,125].

The results obtained from these extensive QTL analyses have potentially significant implications for hop breeding. The patterns of QTL co-location observed in this study (Table 4) suggest that there are separate QTL regions influencing both early and late stages of secondary metabolite biosynthesis. The detection of QTL involved in the early stages of the biosynthetic pathways, either linked or with pleiotropic effects on numerous secondary metabolites, suggests that there is potential for rapid change in the levels of multiple compounds simultaneously; however, the use of these QTL in molecular hop breeding programs may have undesirable consequences. It may be difficult to select for specific secondary metabolites or combinations thereof, without causing a cascade of unpredictable changes to other secondary metabolites. Where the same QTL affects different secondary metabolites relating to opposing objectives, MAS is unlikely to succeed [36]. However, greater confidence can be placed in the specificity of the QTL identified in this study found to influence only a single trait (Table 4; Figure 2). Being compound-specific amongst a large number of secondary metabolites included in this study, these QTL may offer potential to molecular breeding of hop, after validation in further pedigrees and a range of environmental conditions. All of the putative QTL identified present a resource to further our understanding of the genetic basis of variation in traits that influence hop quality (bitterness, flavour and aroma) in beer.

## Conclusions

The QTL analyses conducted in this study revealed several important findings relating to the genetic basis of variation in three issues of relevance to hop breeding programs: expedited plant sex identification, increased yield capacity and improved secondary metabolite profiles, with important implications for the future use of molecular selection methods in hop. We verified a sex-linked marker in a third pedigree; and on the basis of its perfect association with the male sex in this and previous studies [16,22] the HLAGA7 marker would be an effective tool for gender identification of hop plants, a key component of early stage selection in hop breeding programs. We identified an ontogenetically stable QTL for a trait associated with cone yield (dry cone weight). However, for two other metrics of cone yield (green cone weight and harvest index) currently used in routine screening of hop, no QTL were identified. The results for these traits highlight the difficulties of QTL detection for traits which may be controlled by many loci with small effects and for traits under a significant environmental influence. We identified QTL contributing towards explaining the observed phenotypic variation in secondary metabolite accumulation in hop cones through the identification of a QTL for essential oil content. We investigated a total of 45 secondary metabolite traits in this analysis and identified putative QTL affecting 33. The broad range of secondary metabolite traits included in this study provided the first demonstration of extensive pleiotropy/linkage affecting many of these compounds in hop, including many which are apparently unrelated. Pleiotropic/linked loci may present significant complications for molecular breeding, impeding the selection of specific traits without causing undesired alterations to others. In this study, we identified a number of QTL besides the pleiotropic/linked QTL that appeared to be specific to individual secondary metabolites. These QTL potentially offer a direct path to a locus influencing the phenotypic variation of specific secondary metabolites. The linkage maps constructed in this study incorporated a large number of new DArT markers. As DArT markers are transferable, these linkage maps can be employed in other mapping populations, facilitating the identification and validation of further QTL, a crucial step for the broad success of molecular breeding methods in hop. Furthermore, DArT markers can be sequenced to develop more informative co-dominant markers. This study greatly expands our understanding of the complex genetic architecture underlying variation in hop secondary metabolite composition and yield related traits and presents a step forward in hop molecular breeding.

## Methods

### Mapping populations

Two mapping populations were used in this study. Both were F_1_ full-sib families. The first population (New Zealand) consisted of 178 genotypes derived from the cross ‘Nugget’ (female) × ‘Slovenian breeding line (SBL) 3/3’ (male) made in 2005. The population was placed in a randomised order, in rows spaced 2.5 m apart with 1 m between plants within each row. Plants were grown up a 5 m trellis, with 1 string per plant and 2 bines trained up each string. The mapping population was maintained by Plant & Food Research, Motueka, New Zealand. The second population (Slovenian) consisted of 89 individuals derived from the cross ‘Hallertauer Magnum’ (female) × ‘SBL 2/1’ (male) made in 1999. The population was planted in a randomised order, in rows spaced 2.4 m apart with 1.3 m between plants within each row. Plants were grown up a 6.5 m trellis, with 2 strings per plant and 3 bines trained up each string. The mapping population was maintained by the Slovenian Institute of Hop Research and Brewing, Žalec, Slovenia. Both populations were treated with good agronomic practice, taking into consideration optimal fertilisation, irrigation and treatment against diseases and pests (based on prognosis).

### Marker discovery and genotyping

#### DNA extraction

For the development and genotyping of DArT markers, DNA was extracted from the two mapping populations. For the Slovenian population, DNA extraction, as well as the estimation of DNA quality and concentration, was performed as described by Howard et al. [73]. For the New Zealand population, DNA was extracted as described by Buck et al. [19] and treated with RNase A (Life Technologies). DNA was quantified using the Quant-IT™ Broad Range DNA Assay kit on a Qubit fluorometer (Life Technologies). DNA quality was verified by digestion with RsaI. DNA extractions and digests were run on a 1% agarose gel and stained with ethidium bromide for visualisation.

#### DArT marker discovery and genotyping

A first round of DArT marker discovery was conducted in a previous study, whereby 6,144 DArT clones were generated from 92 hop accessions sourced from Europe, Asia, North America and Australia [73]. From these DArT clones, 730 polymorphic markers were identified [73]. A second round of DArT marker discovery was conducted in this study to expand the array and incorporate hop material from New Zealand. DArT markers were developed and their performance evaluated, as described previously [73]. A total of 405 hop accessions were included in the analysis, sourced from New Zealand (186 individuals), Slovenia (93 individuals) and the USA (126 individuals).

A DArT microarray was constructed for the purpose of genotyping the two mapping populations used in this study (from New Zealand and Slovenia) and a third mapping population previously published (from the USA) [17]. The array was composed of markers from both the first and second rounds of markers discovery; only markers that were polymorphic within the mapping populations were included. The microarray was prepared and the two populations genotyped following the method previously described by Howard et al. [73]. DArT genotyping scoring parameters were used to assess marker quality; these parameters included *Q* value, call-rate, reproducibility and polymorphism information content (PIC), as described previously [73].

#### Additional markers for the New Zealand population

An additional 51 markers were used for linkage analysis of the New Zealand population in this study. This included: 43 selected microsatellite markers developed by Brady et al. [126], Jakse et al. [127], Bassil et al. [128], Hadonou et al. [129], Stajner et al. [130], Jakse et al. [131]; four RAPD based markers (Operon Technologies); three STS based markers developed by Danilova and Karlov [20]; and one intron-based DNA marker from the chalcone synthase gene *chs*_H1, produced using the CHSJ5 and CHSJ6 primers developed by Matoušek et al. 2002 [69].

Microsatellite markers were genotyped using either of two methods: independent amplification and visualisation on a CePRO 9600 TM (Combisep, Ames, IA, USA) capillary analysis system, or by undertaking amplification and high resolution melting (HRM) analysis using a Roche Light-Cycler®. Markers screened using the CePRO capillary system were initially amplified in a total volume of 15 μL containing 2 ng of DNA, 0.1 μM of each dNTPs, 1× PCR buffer (Invitrogen), 1.5 mM MgCl_2_, 0.2 μM of each forward and reverse primer, 0.5 U Platinum *Taq*DNA polymerase (Invitrogen). Amplifications were performed in either a 9700 Geneamp Applied Biosystem or a Hybaid MBS 0.5G thermocycler. Initial denaturation at 94°C for 2 min and 30 s was followed by four cycles of 94°C for 30 s, 60°C for 1 min (reduced by 1°C per cycle), 72°C for 1 min, then followed by 30 cycles of 94°C for 30 s, 55°C for 1 min, 72°C for 1 min and a final 5 min 72°C extension. Products were desalted in 96- well microplate UNIFILTER (Whatman, Clifton, NJ, USA) using Sephadex G-75 Superfine (Amersham, Uppsala, Sweden) before analysis on the CePRO capillary system. The alternative HRM genotyping method [132] utilised a 96-well Roche Light-Cycler® 480 (Forester City, CA, USA). Amplification reactions contained 2 ng DNA, 1× Roche master mix, 2.5 mM MgCl_2_ and 0.2 μM of each forward and reverse primer in a 10 μL total volume. These were subject to an initial denaturation step at 95°C for 5 min, followed by four cycles of 95°C for 10 s, 60°C for 30 s (reduced by 1°C per cycle) and 72°C for 15 s; and then 30 cycles of 95°C for 10 s, 55°C for 30 s and 72°C for 15 s. These reactions then underwent the HRM step of 95°C for 1 min (ramp rate 4.4°C/s) with an increase to 65°C (ramp rate 1°C/s) with 25 data acquisitions/°C for 20 min. The melting curves were then analysed using the gene scanning module of the Roche Light-Cycler® 480 collection and analysis software.

RAPD and STS markers were screened following the method outlined for RAPD markers by Buck et al. [133]. Only clear products were scored (fragment size in base pairs is indicated after primer name on linkage map). The chalcone synthase gene based marker (*chs*_H1) was genotyped using the HRM analysis on the Roche Light-Cycler® 480, as outlined above.

#### Additional AFLP and microsatellite markers for the Slovenian population

An additional 241 AFLP markers and 44 microsatellite markers were used for linkage analysis of the Slovenian population in this study. Also included in this study were five markers based on microsatellites within candidate chalcone synthase genes (*vps, chs_*H1, *chs*2, *chs*3 and *chs*4), which encode enzymes directly involved in the biosynthesis of bitter acids [69-71]. These AFLP markers, microsatellite markers and candidate genes have been scored and mapped previously in the Slovenian population [16,79].

### Linkage analysis

A highly stringent linkage analysis method was conducted, using the double pseudo-testcross strategy [134], as in other linkage analyses of hop [14-17]. This was deemed an appropriate strategy, as hop typically displays a high level of heterozygosity [3], and being dioecious, it is the best alternative to a backcross. It is also compatible with DArT markers as they are a dominant marker system [135]. Map construction was carried out using the JoinMap® 4 program [136]. All markers were re-coded by their segregation type according to the cross-pollinated coding scheme (CP) for analysis. Markers were tested for goodness of fit to their assigned Mendelian segregation ratios using the χ^2^ segregation test in JoinMap® 4 [136]. Those markers with significant amounts of segregation distortion (departure from expected Mendelian segregation ratios (α ≥ 0.05)) are indicated with ‘*’ at the end of the locus name (Additional files 1 and 2). Marker type is indicated for each locus at the beginning of the locus name as either ‘D-’ (DArT markers), ‘A-’ (AFLP markers) or ‘S-’ (other marker types) (Additional files 1 and 2). For each population, markers with very low polymorphism (those markers for which one allele was represented by ≤ 10% of the expected scores) and markers with high levels of missing data (≥ 5% of the scores) were eliminated from the analysis. Individuals with high levels of missing data (≥ 10% of the scores) were also eliminated from the analysis.

Separate maternal and paternal linkage maps were constructed from each of the mapping populations, based on the methods described by Keats et al. [137]. Using JoinMap® 4 [136], linkage maps were constructed by grouping significantly associated (linked) markers, statistically estimated through a logarithm (base 10) of odds (LOD) score. Establishing linkage group associations, through the selection of LOD scores is an intuitive process; the theoretical basis for the selection of LOD scores is discussed by Freeman et al. [138]. In this study, linkage groups were generally assigned with a minimum LOD threshold of 4.0, at which the contents of most groups were relatively stable. In unstable groups it was necessary to adjust the LOD threshold to achieve stability. A higher LOD was selected when a linkage group consisted of weakly linked sub-groups, which were eliminated in the process of achieving a stable marker order. The higher LOD threshold allowed the preservation of subgroups, within which there was significant association. A lower LOD was selected when a single marker dropped out of the linkage group at LOD 4.0, in order to maintain as many markers in the analysis as possible.

Within linkage groups, the optimal marker order was determined using JoinMap® 4 [136] default values of a minimum LOD threshold of 2.0, a maximum recombination threshold of 0.35, a maximum χ^2^ goodness-of-fit jump threshold of 5.0 for removal of markers and a ripple value of 1.0. The Kosambi mapping function was used to determine the distance between markers. The linkage phase of markers was determined automatically by the JoinMap® 4 program.

Linkage maps were constructed over several stages. The first stage involved the establishment of a framework map with a reliable marker order, upon which all subsequent analysis was based. This initial analysis was conducted with the highest quality markers, those segregating in a 1:1 ratio that did not show significant segregation distortion. If necessary, markers were removed from the analysis until maps were achieved within two mapping rounds, and all markers had a mean χ^2^ contribution of ≤ 2.0. Markers were removed one at a time, in order of highest mean χ^2^ contribution. Four subsequent stages of analysis were conducted, adding markers to the framework map in the following order of decreasing marker quality: (i) markers segregating in a 1:1 ratio with evidence of segregation distortion; (ii) markers segregating in a 3:1 ratio without evidence of segregation distortion; (iii) markers segregating in a 3:1 ratio with evidence of segregation distortion; and (iv) markers for which the genotype score of one parent was unknown and consequently estimated. At each of these stages of analysis, markers were removed as before, to achieve maps within two mapping rounds and to ensure that all markers had a mean χ^2^ contribution of ≤ 2.0. Markers that contributed to the framework map were not removed and their established marker order was maintained. Markers that instigated a re-ordering of the framework markers were removed. With each subsequent round, markers added to the map in the previous round were not removed and their marker order was maintained. Iterative approaches of adding markers to a framework map, akin to the method used in this analysis, are commonly employed [139-142].

The numbering of linkage groups in all maps followed the numbering established in a previous linkage map of the Slovenian population [16]. Homology between linkage groups was inferred on the basis of shared markers. Where a linkage group was homologous with several linkage groups from the previous linkage map [16], the linkage between these groups was verified at a lower LOD threshold in JoinMap® 4; and the lowest number of the corresponding linkage groups from the previous linkage map of the Slovenian population was assigned. Linkage groups consisting entirely of the newly added markers were assigned the remaining numbers.

Significant clustering of the markers was observed in all maps constructed in this study. For the purpose of QTL analysis, clusters of markers were removed to leave only one marker at each locus (taken as the map position to one decimal place). At the completion of analysis, when all possible markers had been added to the map and the final marker order had been accepted, markers within each cluster were eliminated on the basis of high levels of missing data and then by lower *Q* values (a DArT quality measure). This resulted in between 37 and 73% of the polymorphic markers being removed from the maps.

### Phenotypic measurements

Fifty traits were assessed in hop in this study, related to three issues relevant to the genetic improvement of hop: expediting plant sex identification, increasing yield capacity and improving secondary metabolite composition.

#### Sex trait

Sex was assessed as a binary character by field observation, as either plants bearing male flowers (“0”) or plants bearing female flowers (“1”). Sex of the plants was confirmed over six seasons in the New Zealand population and for at least two years in the Slovenian population.

#### Yield traits

In this study, four yield traits were examined which quantify either the physical yield of cones per plant or the yield of brewing-relevant substance. Three traits assessed cone yield: (i) cone harvest index, a measure of the ratio of fresh or ‘green’ cone weight to the whole plant fresh weight, comparing the allocation of biomass to cone production with the allocation of biomass to vegetative growth; (ii) dry cone weight, a measure of the mass of cones per plant, after the removal of ~95% of the moisture content (leaving a moisture content of 9% by weight of the kiln-dried hop), reflecting the productive vigour of the plant; and (iii) green cone weight, also a measure of the mass of cones per plant, but of the fresh or ‘green’ weight, without consideration of cone moisture content. Cone harvest index and dry cone weight were assessed in the Slovenian population with the aim of identifying ontogenetically stable QTL. Phenotypic measurements were made on every plant in the trial annually over five years, between 2002 and 2006 and the arithmetic mean was calculated from these measurements to give the data used in this analysis. Dry cone weight was quantified according to the EBC 7.2 method for moisture content of hops and hop products [143], as described by Cerenak et al. [16]; cone harvest index was also quantified as described by Cerenak et al. [16]. Green cone weight was assessed in the New Zealand population with the aim of identifying putative QTL. Phenotypic measurements were made in one year, in 2009. The fourth yield trait examined was essential oil content, a measure of the total volume of essential oil secondary metabolites. This trait was examined to determine whether variation in the accumulation of essential oil in hop glandular trichomes has a genetic basis. Essential oil content was measured in the New Zealand population, quantified by steam distillation (see below). Phenotypic measurements were made in one year, in 2009.

The relationship between the two yield traits scored in the Slovenian population (dry cone weight and cone harvest index) and the secondary metabolite trait α-acid (see below) was examined by principal components analysis using the PRINCOMP function in R version 2.11.1 [144]. The first and second vectors accounted for 30% and 10% of the variance, respectively. A correlation matrix was produced, based on Pearson’s product moment correlation coefficients using the COR function (method = “PEARSON”, use = “COMPLETE”) in R version 2.11.1 [144] (n = 3). For the purposes of this investigation, a Pearson’s r value in the range of |0.5| to |0.79| was considered a strong correlation, with |0.8| to |1.0| considered a very strong correlation [145].

#### Secondary metabolite traits

A total of 45 hop cone secondary metabolite traits were assessed in this study (see Additional file 7 for International Union of Pure and Applied Chemistry (IUPAC) names of chemical compounds), from all significant hop secondary metabolite groups (hop acids, essential oils and polyphenols). All secondary metabolite traits were assessed in the New Zealand population, with α-acid also assessed in the Slovenian population. In the New Zealand population, phenotypic measurements were made in one year, in 2009, with the aim of identifying putative QTL. In the Slovenian population, the aim was to identify environmentally and ontogenetically stable QTL and as such, phenotypic measurements were made annually over five years, between 2002 and 2006 and the data averaged. With the exception of α-acid, none of the secondary metabolite traits have been previously assessed in either the New Zealand or Slovenian populations. Hop acids comprise both α- and β-acids; a total of nine traits relating to hop acids were quantified in this study: (i) α-acid content; (ii) β-acid content; (iii) the ratio of α-acid to β-acid; (iv) the percentage of α-acid that is cohumulone (a major constituent of α-acid); (v) the percentage of β-acid that is colupulone (a major constituent of β-acid); (vi) cohumulone content; (vii) colupulone content; (viii) humulone + adhumulone (the other major constituents of α-acid) content; and (ix) lupulone + adlupulone (the other major constituents of the β-acid) content. Essential oils comprise oxygenated compounds (esters, ketones, ethers, monoterpene alcohols and sesquiterpene alcohols) and terpenoid compounds (monoterpenes and sesquiterpenes). A total of 33 essential oil compounds were assessed in this study; these were five esters (methyl-4-methylhex-2-enoate, methyl dec-4-enoate, methyl decanoate geranyl acetate and geranyl isobutyrate), one ketone (2undecanone), four ethers (humulene diepoxide a, humulene epoxide I, II and III), three monoterpene alcohols (geraniol, linalool and limonene-10-ol), four sesquiterpene alcohols (caryolan-1-ol, humulenol II, humulol and t-cadinol), one alkane (tetradecane), six monoterpenes (β-pinene, camphene, limonene, myrcene, ρ-cymene, terpinene) and 10 sesquiterpenes (α-capaene, α-selinene, β-selinene, δ-cadinene, γ-cadinene, caryophyllene oxide, caryophyllene, farnesene, humulene, muurolene). The ratio of humulene to caryophyllene was also assessed, as it is a reliable maturity indicator [3] and is often used for varietal characterisation. One polyphenol, xanthohumol, was scored in this study.

The relationships among the 45 hop secondary metabolite traits assessed in the New Zealand population were examined using principal components analysis. The first and second vectors accounted for 55% and 26% of the total variance, respectively. A correlation matrix was produced, as described above (n = 47).

The hop acid and polyphenol components of the cone secondary metabolite profile of the New Zealand population were analysed by HPLC. Extracts were prepared in 2009, by grinding 10 g hop cone tissue with 100 mL toluene using an Omni Macro ES homogeniser (Omni International, Marietta, GA) then filtered. A volume of 3 ml of the filtrate was added to 47 ml methanol and inverted four times. The extracts were fractioned by HPLC, on a system consisting of a Shimadzu LC 6A/LC 10AS pump, a Shimadzu SIL-10AF autosampler (10 μL sample loop) and a UV/UV–vis Shimadzu SPD 10A detector at a wavelength of 314 nm. A Kinetix reversed-phase C18 column (100 × 4.6 mm; 2.6 μm particle size) was used (Phenomenex, Torrance, CA, USA), heated to 30°C with a Shimadzu CTO 10A column oven. The mobile phase used for separation was a methanol–water-phosphoric acid mixture (in a ratio of 85:17:0.25 V/V/V), at a flow rate of 1.2 ml/min, for 16 min. The sample volume injected was 10 μL. A Shimadzu LC Solution software package was used for quantification. Standardised hop extract (ICE-3) with known content of α- and β-acids and xanthohumol were injected for identification and quantitative analysis, and their retention times and spectra compared. Five components (xanthohumol, cohumulone, n + adhumulone, colupulone, n + adlupulone) were identified and quantified, with other traits derived by calculation from these five components (α-acid = cohumulone + (n + adhumulone); β-acid = colupulone + (n + adlupulone); percentage of α-acid that is cohumulone = cohumulone/α-acid; percentage of β-acid that is colupulone = colupulone/β-acid; ratio of α-acid to β-acid = α-acid/β-acid).

The essential oil content of harvested cones from the New Zealand population was estimated by steam distillation, following the EBC 7.10 method for hop oil content of hops and hop products [143]; and the individual essential oil components of the cone secondary metabolite profile were analysed by GCFID on a Shimadzu GC-2010 system fitted with an AOC20i autosampler. Essential oil extracts were prepared in 2009 by steam-distillation of 100 g of ground hop cone tissue. A volume of 100 μL of essential oil was added to 1 ml of double distilled diethyl ether for GC analysis. The extracts were fractioned by GCFID, using Shimadzu GC Solution software. Each of the 33 essential oil components targeted and quantified in this analysis were expressed as the percentage of their peak area to the total area of all essential oil peaks eluted. The ratio of humulene to caryophyllene was additionally calculated.

The hop acid trait α-acid was also measured in the Slovenian population, analysed by the lead conductance value (LCV) measure, following the EBC 7.4 method for LCV of hops, powders and pellets [143], as described by Cerenak et al. [16]. Although obtained through different extraction and quantification methods, α-acid content as assessed in the Slovenian population is analogous to α-acid content as assessed in the New Zealand population, allowing direct comparison of this trait across the two separate experiments.

### QTL analysis

QTL analysis was conducted using the linkage maps constructed in this study. MapQTL® 6 [146] was used for this analysis. Putative QTL were declared at the genome-wide significance level (α < 0.05). The LOD threshold for genome-wide significance was estimated by permutation testing with 10000 iterations [147]. This method determines the LOD threshold for each phenotypic trait separately and, unlike other empirical methods, makes no assumptions regarding probability distribution [147]. Interval mapping (IM) was conducted, using the regression algorithm and the default MapQTL® 6 parameters [146], to scan the genome for map intervals significantly associated with traits. Where map intervals exceeded the genome-wide LOD threshold, single markers with the highest LOD value were selected as cofactors for multiple QTL model (MQM) mapping. MQM mapping was performed using an iterative approach with the forward selection of cofactors until a stable set of cofactors was established.

Due to the high proportion of dominant markers (in linkage groups where markers are segregating from one parent only), MapQTL® 6 [146] was unable to reach a unique solution to the probability of the QTL genotype due to the existence of more than one solution to the set of mathematical equations, as described by Van Ooijen [146]. To overcome this problem, the two-way pseudo-testcross analysis was undertaken, whereby the marker data was separated into the two meioses (markers segregating from respective parents only) and recoded from the CP population type to the doubled haploid population type (DH), as described by Van Ooijen [146]. IM and MQM then proceeded again, as described above.

Identified QTL were confirmed with single marker non-parametric Kruskal-Wallis (KW) testing (P < 0.05). KW testing is a particularly robust calculation in cases where the distribution of a trait departs from normality [146]. KW testing was also used to determine whether the QTL was segregating from the male or female parent.

Male and female maps for each population were drawn using MapChart® 2.2 [148]. The QTL identified were indicated with solid bars representing 1-LOD support intervals and lines representing a 2-LOD support intervals. The 2-LOD support interval corresponds to an ~95% confidence interval [149].

## Abbreviations

AFLP: Amplified fragment length polymorphism; CP: Cross-pollinated; DArT: Diversity arrays technology; DH: Doubled haploid; DMAPP: Dimethylallyl diphosphate; Gbp: Giga base pairs; HRM: High resolution melting; HPLC: High-performance liquid chromatography; ISSR: Inter simple sequence repeat; IM: Interval mapping; IPP: Isopentenyl pyrophosphate; IUPAC: International union of pure and applied chemistry; KW: Kruskal-Wallis; LCV: Lead conductance value; LG: Linkage group; LOD: Logarithm (base 10) of odds; MAS: Marker assisted selection; MEP: 2-C-methylerythritol 4-phosphate; MQM: Multiple QTL model; pg: Picograms; PIC: Polymorphism information content; QTL: Quantitative trait loci; RAPD: Random amplification of polymorphic DNA; SBL: Slovenian breeding line; SSR: Simple sequence repeat; STS: Sequence-tagged site.

## Competing interests

Employees of DArT PL co-authoring this paper (AKi and JC) provide DArT array commercial genotyping services for a range of crops and may benefit financially from this work.

## Authors’ contributions

EM performed linkage, correlation and QTL analyses, interpreted the data and wrote the manuscript. JF assisted with linkage and QTL analyses, assisted with data interpretation and revised the manuscript. SW assisted with correlation analysis and revised the manuscript. EB, with help from CHW, provided DNA for DArT marker development and DArT genotyping of the New Zealand mapping population, obtained the genotypic data for the non-DArT markers used in the New Zealand mapping population of this study and revised the manuscript. DA contributed the chemical data for the New Zealand mapping population and revised the manuscript. LG and DG provided the sex phenotypic data for the New Zealand mapping population. RB developed the New Zealand mapping population. AC, JJ and BJ provided DNA for DArT marker development. AC provided the phenotypic and chemical data for the Slovenian mapping population. JJ provided DNA for DArT genotyping of the Slovenian mapping population. BJ revised the manuscript. JC and AKi performed the DArT marker discovery analysis, developed the DArT array and genotyped the two mapping populations with the DArT markers. PD contributed to DArT marker development. RV assisted with data interpretation and revised the manuscript. AKo led the international hop DArT collaboration and was the instigator and co-ordinator of the study. All authors were involved in the conception of the study and read and approved the final manuscript.

## Supplementary Material

Additional file 1 **Maternal linkage map of the New Zealand hop mapping population.** There is evidence from homology to the linkage map of a Slovenian mapping population also constructed in this study that two linkage groups from the maternal ‘Nugget’ map can be linked to form ‘Nugget’ LG1. In our study, these linkage groups form one group at LOD 2. Ten linkage groups (seven major, one triplet and two doublets) were identified in the maternal ‘Nugget’ map. Click here for file

Additional file 2 **Paternal linkage map of the New Zealand hop mapping population.** Eight linkage groups (five major, one triplet and two doublets) were identified in the paternal ‘S.B.L. 3/3’ map. Click here for file

Additional file 3 **Linkage group homology between maternal and paternal linkage maps of the New Zealand and Slovenian populations and between linkage maps of the Slovenian population constructed in this study and linkage maps of the Slovenian population constructed in a previous study **[16]**.** Click here for file

Additional file 4 **Maternal linkage map of the Slovenian hop mapping population.** There is evidence from homology to a previous linkage map of the Slovenian mapping population [16] that several of the linkage groups from the maternal linkage map map can be linked. The two linkage groups that link to form ‘Hallertauer Magnum’ LG1 in this study form one group at LOD 5. The three linkage groups that link to form ‘Hallertauer Magnum’ LG2 in this study form one group at LOD 4. The two linkage groups that link to form ‘Hallertauer Magnum’ LG4 in this study form one group at LOD 3. Ten linkage groups were identified in the maternal ‘Nugget’ map (eight major and two doublets). Click here for file

Additional file 5 **Paternal linkage map of the Slovenian hop mapping population.** There is evidence from homology to a previous linkage map of the Slovenian mapping population [16] that two linkage groups from the paternal linkage map can be linked. The two linkage groups that link to form ‘S.B.L. 2/1’ LG1 in this study form one group at LOD 4. Ten linkage groups were identified in the paternal ‘S.B.L. 2/1’ map (six major, one triplet and three doublets). Click here for file

Additional file 6 Correlations between sex, yield and secondary metabolite traits affected by each pleiotropic/linked locus identified in hop. Click here for file

Additional file 7 International Union of Pure and Applied Chemistry (IUPAC) naming of secondary metabolites quantified in hop. Click here for file
