# Supplementary material for: Quantitative trait loci in hop (Humulus lupulus L.) reveal complex genetic architecture underlying variation in sex, yield and cone chemistry
Source: BMC Genomics. 2013 May 30;14:360. doi: 10.1186/1471-2164-14-360 (PMC3680207; doi:10.1186/1471-2164-14-360)
Supplement: Additional file 3 — Linkage group homology between maternal and paternal linkage maps of the New Zealand and Slovenian populations and between linkage maps of the Slovenian population constructed in this study and linkage maps of the Slovenian population constructed in a previous study [16]. [file 1471-2164-14-360-S3.docx]

| Linkage map of New Zealand population, this study | | Linkage map of Slovenian population, this study | | Linkage map of Slovenian population, previous study | |
| --- | --- | --- | --- | --- | --- |
| Nugget ♀ | S.B.L. 3/3 ♂ | Hallertauer Magnum ♀ | S.B.L. 2/1 ♂ | Hallertauer Magnum ♀ | S.B.L. 2/1 ♂ |
| 1a^2^ |  | 1a^2,3,4^ | 1a^3,4^ | 1^4^ | 1^4^ |
| 1b^1,2^ | 1^1,2^ | 1b^2,3,4^ | 1b^2,3,4^ | 1+11^4^ | 1^4^ |
| 2^2^ | 2 | 2a^2,3,4^ | 2^3,4^ | 2+10+12^4^ | 2^4^ |
|  |  | 2b^4^ |  | 2+9^4^ |  |
|  |  | 2c^4^ |  | 2^4^ |  |
| 3^2^ | 3^2^ | 3^2,3,4^ | 3^2,3,4^ | 3^4^ | 3^4^ |
| 4^1,2^ | 4^1,2^ | 4a^2,4^ | 4^2^ | 4^4^ | 4 |
|  |  | 4b^4^ |  | 4^4^ |  |
| 5^1,2^ | 5^1,2^ | 5^2,4^ | 5^2,4^ | 5^4^ | 5+15+18^4^ |
| 6^1^ | 6^1,2^ | 6^3,4^ | 6^2,3,4^ | 6^4^ | 6^4^ |
| 7^1^ | 7^1^ | 7^4^ | 7^4^ | 7+9^4^ | 7^4^ |
| 8^1^ | 8^1^ | 8^4^ | 8^4^ | 8^4^ | 19^4^ |
| 9 |  | 9^4^ | 9^4^ | 14^4^ | 17^4^ |
| 10 |  | 10 | 10 |  | 16 |
|  |  |  |  | 13 | 20 |

1 = match between Nugget (this study) and S.B.L. 3/3 (this study)

2 = match between New Zealand population (this study) and Slovenian population (this study)

3 = match between Hallertauer Magnum (this study) and S.B.L. 2/1 (this study)

4 = match between Slovenian population (this study) and Slovenian population (previous study)
