# Supplementary material for: Quantitative trait loci in hop (Humulus lupulus L.) reveal complex genetic architecture underlying variation in sex, yield and cone chemistry
Source: BMC Genomics. 2013 May 30;14:360. doi: 10.1186/1471-2164-14-360 (PMC3680207; doi:10.1186/1471-2164-14-360)
Supplement: Additional file 7 — International Union of Pure and Applied Chemistry (IUPAC) naming of secondary metabolites quantified in hop. [file 1471-2164-14-360-S7.docx]

| **chemical group** | | trait name | IUPAC name |
| --- | --- | --- | --- |
| hop acid | α-acid | humulone | (6*R*)-3,5,6-Trihydroxy-2-(3-methylbutanoyl)-4,6-bis(3-methylbut-2-en-1-yl)cyclohexa-2,4-dien-1-one |
|  |  | adhumulone | 3,5,6-trihydroxy-2-(2-methylbutanoyl)-4,6-bis(3-methylbut-2-enyl)cyclohexa-2,4-dien-1-one |
|  |  | cohumulone | 3,5,6-trihydroxy-4,6-bis(3-methylbut-2-enyl)-2-(2-methylpropanoyl)cyclohexa-2,4-dien-1-one |
|  | β-acid | lupulone | 3,5-dihydroxy-2-(3-methylbutanoyl)-4,6,6-tris(3-methylbut-2-enyl)cyclohexa-2,4-dien-1-one |
|  |  | adlupulone | 3,5-Dihydroxy-2,6,6-tris(3-methyl-2-butenyl)-4-(2-methyl-1-oxobutyl)-2,4-cyclohexadien-1-one |
|  |  | colupulone | 3,5-dihydroxy-4,6,6-tris(3-methylbut-2-enyl)-2-(2-methylpropanoyl)cyclohexa-2,4-dien-1-one |
| essential oil | ester | geranyl acetate | [(2E)-3,7-dimethylocta-2,6-dienyl] acetate |
|  |  | geranyl isobutyrate | (2E)-3,7-dimethylocta-2,6-dienyl] 2-methylpropanoate |
|  |  | methyl decanoate | methyl decanoate |
|  |  | methyl dec-4-enoate | methyl dec-4-enoate |
|  |  | methyl-4-methylhex-2-enoate | (E)-4-methylhex-2-enoate |
|  | ketone | 2-undecanone | Undecan-2-one |
|  | ether | humulene diepoxide a | 6,7-diepoxy-9-[2,6,6,9-tetramethylcycloundecatriene] |
|  |  | humulene epoxide I | (4E,7E,11R)-1,5,9,9-tetramethyl-12-oxabicyclo[9.1.0]dodeca-4,7-diene |
|  |  | humulene epoxide II | (3E,7E,11R)-1,5,5,8-tetramethyl-12-oxabicyclo[9.1.0]dodeca-3,7-diene |
|  |  | humulene epoxide III | (3E,7E)-3,7,10,10-tetramethyl-12-oxabicyclo[9.1.0]dodeca-3,7-diene |
|  | monoterpene alcohol | geraniol | (2E)-3,7-dimethylocta-2,6-dien-1-ol |
|  |  | limonene-10-ol | 2-[(1R)-4-methyl-1-cyclohex-3-enyl]prop-2-en-1-ol |
|  |  | linalool | 3,7-dimethylocta-1,6-dien-3-ol |
|  | sesquiterpene  alcohol | caryolan-1-ol | (1*S*,2*R*,5*S*,8*R*)-4,4,8-trimethyltricyclo[6.3.1.0]dodecan-1-ol |
|  |  | humulenol II | (1R,4E,8E)-6,6,9-Trimethyl-2-methylene-4,8-cycloundecadien-1-ol |
|  |  | humulol | humulol |
|  |  | t-cadinol | (1S,4S,4aR,8aR)-1,6-dimethyl-4-propan-2-yl-3,4,4a,7,8,8a-hexahydro-2H-naphthalen-1-ol |
|  | alkane | tetradecane | tetradecane |
|  | monoterpene | β-pinene | 7,7-dimethyl-4-methylidenebicyclo[3.1.1]heptane |
|  |  | camphene | 6,6-dimethyl-5-methylidenebicyclo[2.2.1]heptane |
|  |  | limonene | 1-methyl-4-(1-methylethenyl)-cyclohexene |
|  |  | myrcene | 7-methyl-3-methylideneocta-1,6-diene |
|  |  | ρ-cymene | 1-methyl-4-propan-2-ylbenzene |
|  |  | terpinene | 1-methyl-4-propan-2-ylcyclohexa-1,3-diene |
|  | sesquiterpene | α-copaene | 8-isopropyl-1,3-dimethyl tricycle(4.4.0.02,7)dec-3-ene |
|  |  | α-selinene | 5,8a-dimethyl-3-prop-1-en-2-yl-2,3,4,4a,7,8-hexahydro-1H-naphthalene |
|  |  | β-selinene | (3S,4aR,8aS)-8a-methyl-5-methylidene-3-prop-1-en-2-yl-1,2,3,4,4a,6,7,8-octahydronaphthalene |
|  |  | δ-cadinene | 4,7-dimethyl-1-propan-2-yl-1,2,3,5,6,8a-hexahydronaphthalene |
|  |  | γ-cadinene | 7-methyl-4-methylidene-1-propan-2-yl-2,3,4a,5,6,8a-hexahydro-1H-naphthalene |
|  |  | caryophyllene | (4Z)-4,11,11-trimethyl-8-methylenebicyclo(7.2.0)undec-4-ene |
|  |  | caryophyllene oxide | [1R-(1R*,4R*,6R*,10S*)]-4,12,12-trimethyl-9-methylene-5-oxatricyclo[8.2.0.04,6]dodecane |
|  |  | farnesene | (3E,6E)-3,7,11-trimethyldodeca-1,3,6,10-tetraene |
|  |  | humulene | 2,6,6,9-tetramethylcycloundecatriene |
|  |  | muurolene | (1S,4aS,8aR)-1-isopropyl-4,7-dimethyl-1,2,4a,5,6,8a-hexahydronaphthalene |
| poly-  phenol | poly-  phenol | xanthohumol | (E)-1-[2,4-dihydroxy-6-methoxy-3-(3-methylbut-2-enyl)phenyl]-3-(4-hydroxyphenyl)prop-2-en-1-one |
