# Supplementary material for: Quantitative trait loci in hop (Humulus lupulus L.) reveal complex genetic architecture underlying variation in sex, yield and cone chemistry
Source: BMC Genomics. 2013 May 30;14:360. doi: 10.1186/1471-2164-14-360 (PMC3680207; doi:10.1186/1471-2164-14-360)
Supplement: Supplementary file 3 [file 1471-2164-14-360-S1.docx]

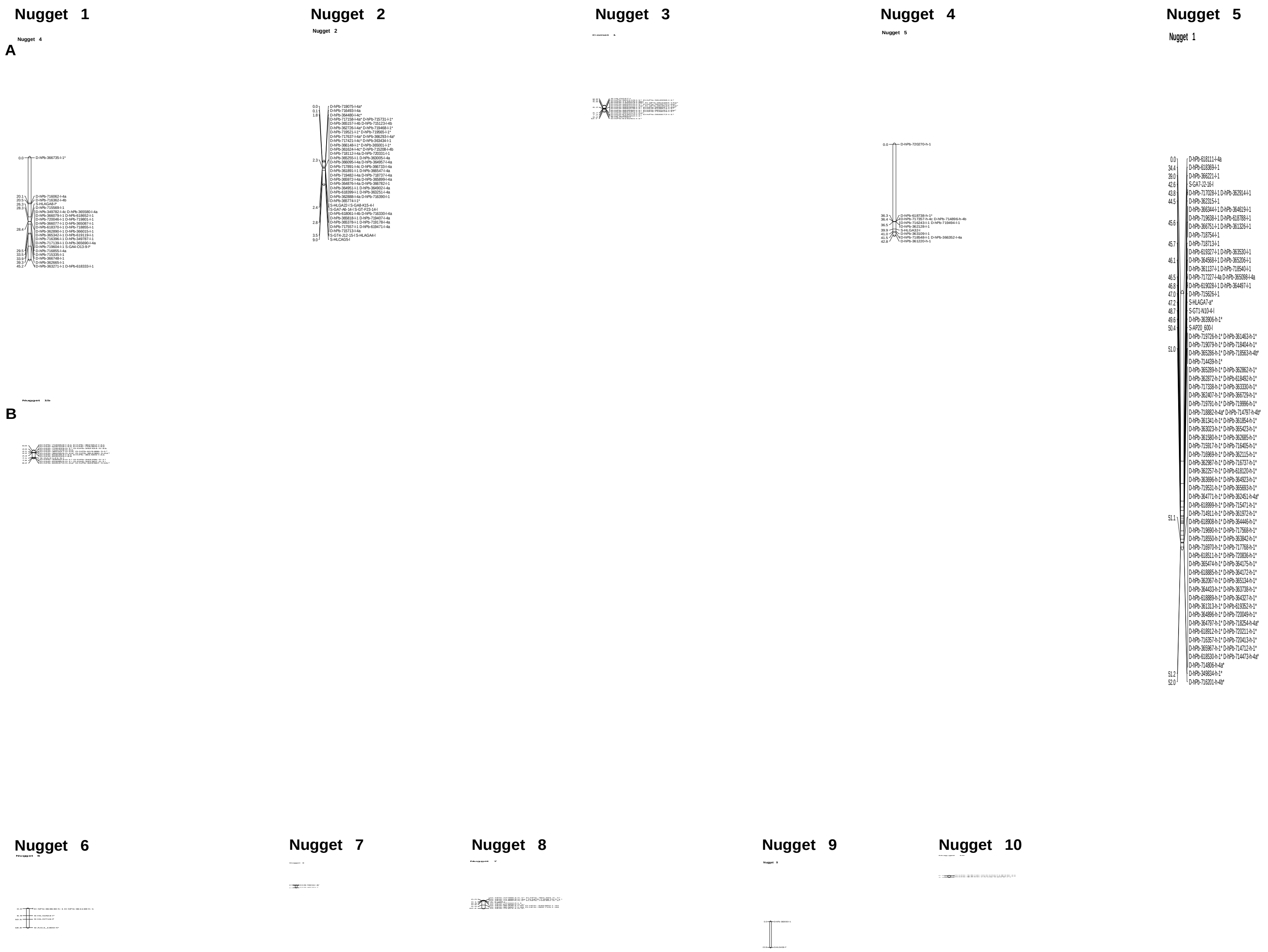
Nugget 1
Nugget 2
Nugget 3
Nugget 4
Nugget 5
A
B
Nugget 6
Nugget 7
Nugget 8
Nugget 9
Nugget 10
