# Supplementary material for: Quantitative trait loci in hop (Humulus lupulus L.) reveal complex genetic architecture underlying variation in sex, yield and cone chemistry
Source: BMC Genomics. 2013 May 30;14:360. doi: 10.1186/1471-2164-14-360 (PMC3680207; doi:10.1186/1471-2164-14-360)

S.B.L. 3/3 1

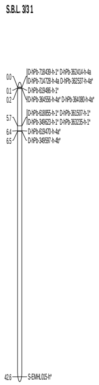

S.B.L. 3/3 2

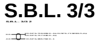

S.B.L. 3/3 3

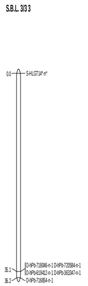

S.B.L. 3/3 4

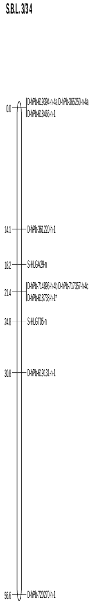

S.B.L. 3/3 5

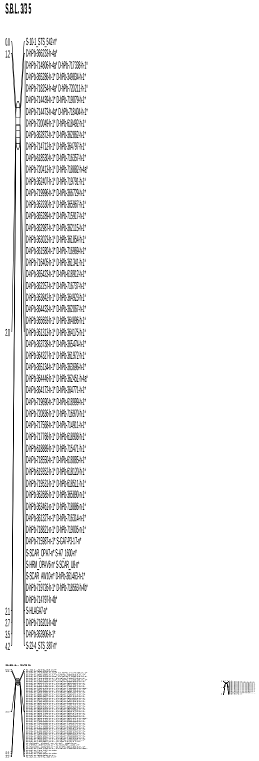

S.B.L. 3/3 6

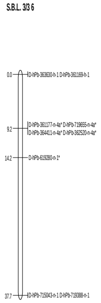

S.B.L. 3/3 7

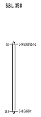

S.B.L. 3/3 8

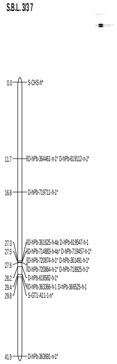

Supplement: Supplementary file 4 [file 1471-2164-14-360-S2.docx]
