# Supplementary material for: Quantitative trait loci in hop (Humulus lupulus L.) reveal complex genetic architecture underlying variation in sex, yield and cone chemistry
Source: BMC Genomics. 2013 May 30;14:360. doi: 10.1186/1471-2164-14-360 (PMC3680207; doi:10.1186/1471-2164-14-360)
Supplement: Supplementary file 5 [file 1471-2164-14-360-S4.docx]

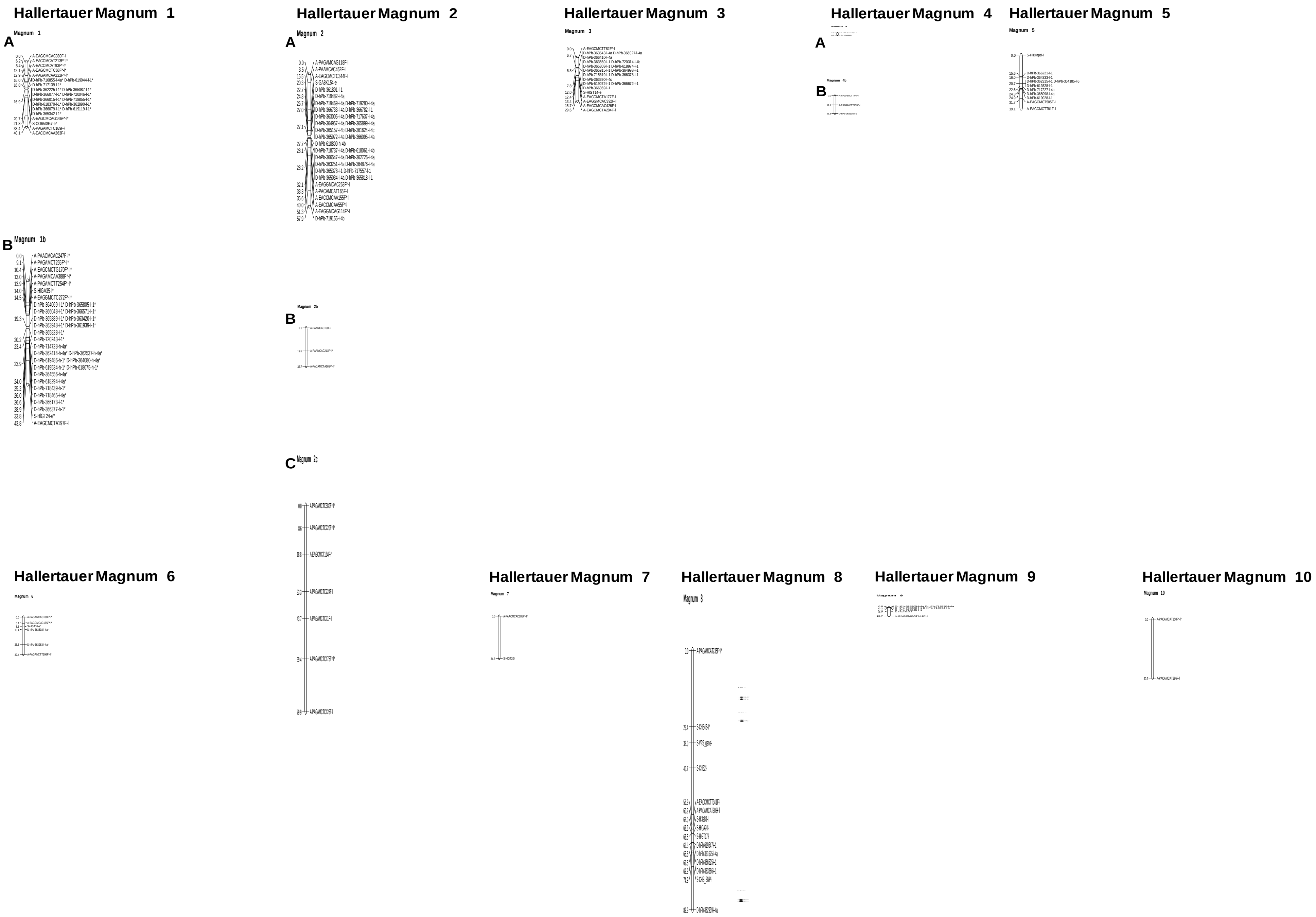

Hallertauer Magnum 1
A
Magnum 1
B
Magnum 1b
Hallertauer Magnum 2
A
Magnum 2
B
Magnum 2b
C
Magnum 2c
Hallertauer Magnum 3
Magnum 3
Hallertauer Magnum 4
A
B
Magnum 4b
Hallertauer Magnum 5
Magnum 5
Hallertauer Magnum 6
Magnum 6
Hallertauer Magnum 7
Magnum 7
Hallertauer Magnum 8
Magnum 8
Hallertauer Magnum 9
Magnum 9
Hallertauer Magnum 10
Magnum 10
