# Supplementary material for: Quantitative trait loci in hop (Humulus lupulus L.) reveal complex genetic architecture underlying variation in sex, yield and cone chemistry
Source: BMC Genomics. 2013 May 30;14:360. doi: 10.1186/1471-2164-14-360 (PMC3680207; doi:10.1186/1471-2164-14-360)
Supplement: Supplementary file 6 [file 1471-2164-14-360-S5.docx]

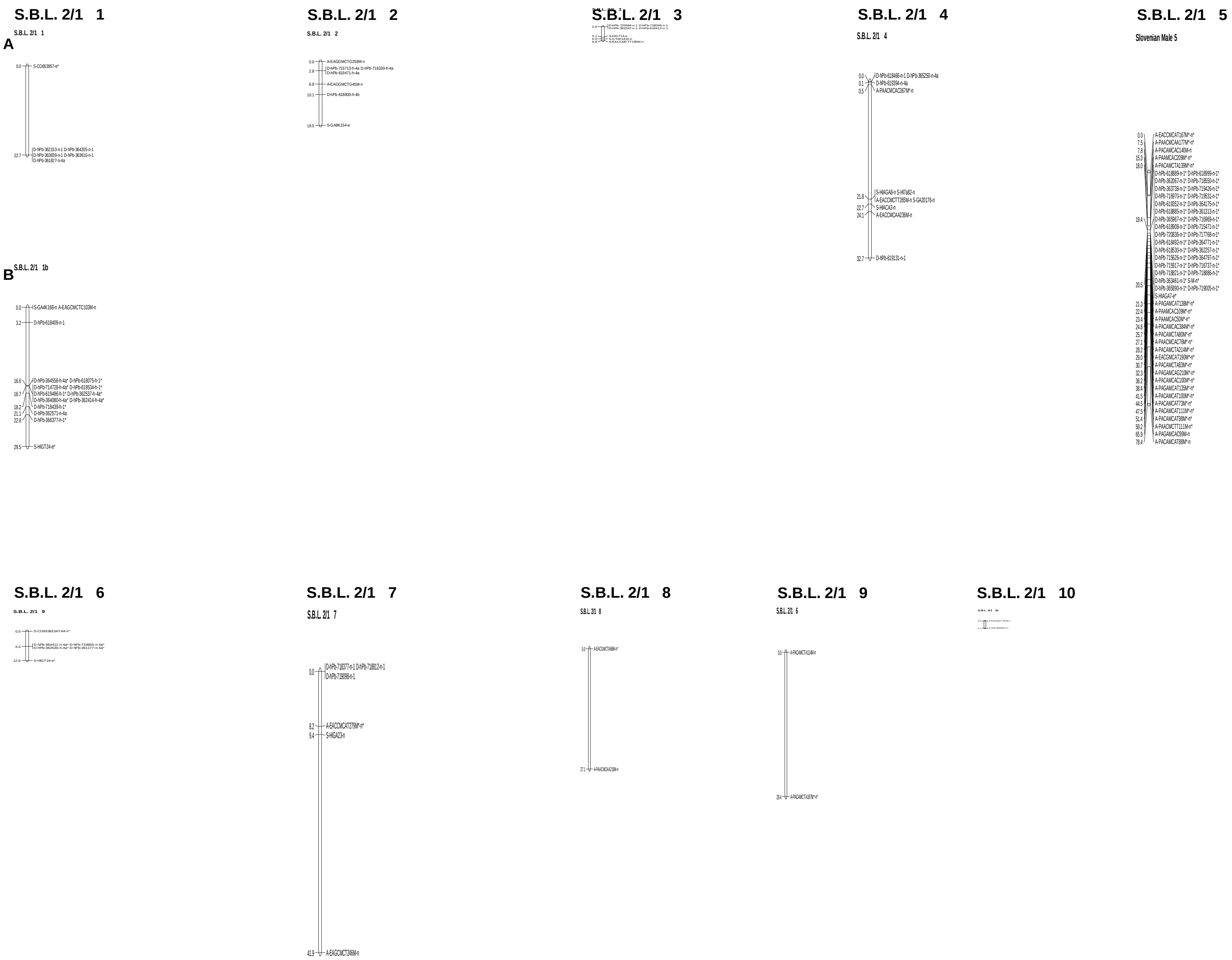
S.B.L. 2/1 1
A
B
S.B.L. 2/1 2
S.B.L. 2/1 3
S.B.L. 2/1 4
S.B.L. 2/1 5
Slovenian Male 5
S.B.L. 2/1 6
S.B.L. 2/1 7
S.B.L. 2/1 8
S.B.L. 2/1 9
S.B.L. 2/1 10
