# Supplementary material for: Quantitative trait loci in hop (Humulus lupulus L.) reveal complex genetic architecture underlying variation in sex, yield and cone chemistry
Source: BMC Genomics. 2013 May 30;14:360. doi: 10.1186/1471-2164-14-360 (PMC3680207; doi:10.1186/1471-2164-14-360)
Supplement: Supplementary file 7 [file 1471-2164-14-360-S6.docx]

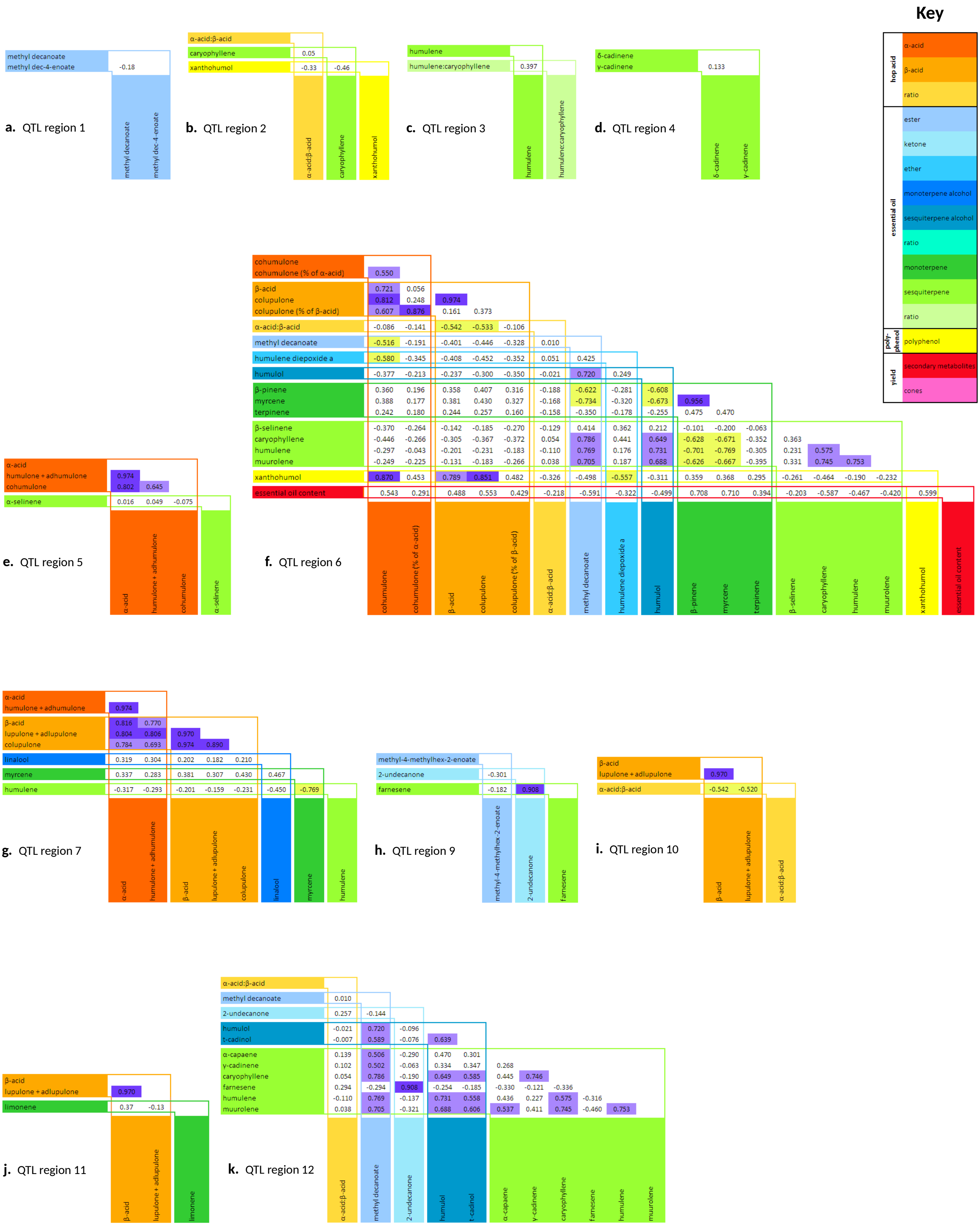
Key
hop acid: α-acid, β-acid, ratio
essential oil: ester, ketone, ether, monoterpene alcohol, sesquiterpene alcohol, ratio, monoterpene, sesquiterpene, ratio
polyphenol: polyphenol
yield: secondary metabolites, cones
a. QTL region 1
methyl decanoate
methyl dec-4-enoate -0.18
b. QTL region 2
α-acid:β-acid
caryophyllene 0.05
xanthohumol -0.33 -0.46
c. QTL region 3
humulene
humulene:caryophyllene 0.397
d. QTL region 4
δ-cadinene
γ-cadinene 0.133
e. QTL region 5
α-acid
humulone + adhumulone 0.974
cohumulone 0.802 0.645
α-selinene 0.016 0.049 -0.075
f. QTL region 6
cohumulone
cohumulone (% of α-acid) 0.550
β-acid 0.721 0.056
colupulone 0.812 0.248 0.974
colupulone (% of β-acid) 0.607 0.876 0.161 0.373
α-acid:β-acid -0.086 -0.141 -0.542 -0.533 -0.106
methyl decanoate -0.516 -0.191 -0.401 -0.446 -0.328 0.010
humulene diepoxide a -0.580 -0.345 -0.408 -0.452 -0.352 0.051 0.425
humulol -0.377 -0.213 -0.237 -0.300 -0.350 -0.021 0.720 0.249
β-pinene 0.360 0.196 0.358 0.407 0.316 -0.188 -0.622 -0.281 -0.608
myrcene 0.388 0.177 0.381 0.430 0.327 -0.168 -0.734 -0.320 -0.673 0.956
terpinene 0.242 0.180 0.244 0.257 0.160 -0.158 -0.350 -0.178 -0.255 0.475 0.470
β-selinene -0.370 -0.264 -0.142 -0.185 -0.270 -0.129 0.414 0.362 0.212 -0.101 -0.200 -0.063
caryophyllene -0.446 -0.266 -0.305 -0.367 -0.372 0.054 0.786 0.441 0.649 -0.628 -0.671 -0.352 0.363
humulene -0.297 -0.043 -0.201 -0.231 -0.183 -0.110 0.769 0.176 0.731 -0.701 -0.769 -0.305 0.231 0.575
muurolene -0.249 -0.225 -0.131 -0.183 -0.266 0.038 0.705 0.187 0.688 -0.626 -0.667 -0.395 0.331 0.745 0.753
xanthohumol 0.870 0.453 0.789 0.851 0.482 -0.326 -0.498 -0.557 -0.311 0.359 0.368 0.295 -0.261 -0.464 -0.190 -0.232
essential oil content 0.543 0.291 0.488 0.553 0.429 -0.218 -0.591 -0.322 -0.499 0.708 0.710 0.394 -0.203 -0.587 -0.467 -0.420 0.599
cohumulone, cohumulone (% of α-acid), β-acid, colupulone, colupulone (% of β-acid), α-acid:β-acid, methyl decanoate, humulene diepoxide a, humulol, β-pinene, myrcene, terpinene, β-selinene, caryophyllene, humulene, muurolene, xanthohumol, essential oil content
g. QTL region 7
α-acid
humulone + adhumulone 0.974
β-acid 0.816 0.770
lupulone + adlupulone 0.804 0.806 0.970
colupulone 0.784 0.693 0.974 0.890
linalool 0.319 0.304 0.202 0.182 0.210
myrcene 0.337 0.283 0.381 0.307 0.430 0.467
humulene -0.317 -0.293 -0.201 -0.159 -0.231 -0.450 -0.769
h. QTL region 9
methyl-4-methylhex-2-enoate
2-undecanone -0.301
farnesene -0.182 0.908
i. QTL region 10
β-acid
lupulone + adlupulone 0.970
α-acid:β-acid -0.542 -0.520
j. QTL region 11
β-acid
lupulone + adlupulone 0.970
limonene 0.37 -0.13
k. QTL region 12
α-acid:β-acid
methyl decanoate 0.010
2-undecanone 0.257 -0.144
humulol -0.021 0.720 -0.096
t-cadinol -0.007 0.589 -0.076 0.639
α-capaene 0.139 0.506 -0.290 0.470 0.301
γ-cadinene 0.102 0.502 -0.063 0.334 0.347 0.268
caryophyllene 0.054 0.786 -0.190 0.649 0.585 0.445 0.746
farnesene 0.294 -0.294 0.908 -0.254 -0.185 -0.330 -0.121 -0.336
humulene -0.110 0.769 -0.137 0.731 0.558 0.436 0.227 0.575 -0.316
muurolene 0.038 0.705 -0.321 0.688 0.606 0.537 0.411 0.745 -0.460 0.753
